# Oomycetes manipulate plant innate immunity through galacturonide oxidases

**DOI:** 10.1038/s41467-025-64189-1

**Published:** 2025-10-20

**Authors:** Lydia R. J. Welsh, Anna O. Avrova, Katrin Besser, Talia Kirkbride, Carla Botelho Machado, Natasha E. Hatton, Leonardo D. Gomez, Martin A. Fascione, Jared Cartwright, Petra C. Boevink, Katherine Denby, David Cannella, Simon J. McQueen-Mason, Stephen C. Whisson, Federico Sabbadin

**Affiliations:** 1 Cell and Molecular Sciences, James Hutton Institute, Invergowrie, Dundee, UK; 2 Centre for Novel Agricultural Products, Department of Biology, University of York, York, UK; 3 Department of Chemistry, University of York, York, UK; 4 Bioscience Technology Facility, Department of Biology, University of York, York, UK; 5 PhotoBiocatalysis Unit, Crop Production and Biostimulation Lab, Universitè libre de Bruxelles, Brussels, Belgium

**Keywords:** Carbohydrates, Pathogens, Virulence, Cellular microbiology, Oxidoreductases

## Abstract

*Phytophthora infestans* is a damaging crop pathogen and a model oomycete for studying plant-pathogen interactions. We report the functional characterisation of a group of *P. infestans* berberine bridge enzyme-like proteins (BBEs) and their role in plant infection. We demonstrate that BBE-encoding genes are upregulated early during infection and that the secreted enzymes specifically oxidise fragments of pectin, the most abundant charged polysaccharide in the plant cell wall. We further show that these enzymes preferentially oxidise longer pectin fragments, which evade detection by the plant and fail to trigger reactive oxygen species (ROS) signalling. Microscopy revealed that the most abundant *P. infestans* BBE localises at germ tube tips prior to leaf penetration, and at haustoria during early infection. Combined with the reduced infection observed upon silencing of the encoding genes, these findings point to a key role for this enzyme class in host penetration and colonisation by microbial pathogens. The identification of BBEs as oomycete pathogenicity factors opens new opportunities for crop protection and food security.

## Abstract (web summary)

The oomycete *Phytophthora infestans* is a damaging crop pathogen. Here, the authors show that a group of *P. infestans* secreted enzymes play roles in penetration and colonization of host plants by oxidising fragments of the polysaccharide pectin in the plant cell wall.

## Introduction

Together, fungi and oomycetes are the most damaging pathogens in modern agriculture and represent a persistent threat to global food security^1^. The oomycete *Phytophthora* genus alone contains over 200 species^2^, including the potato and tomato late blight pathogen *Phytophthora infestans*. This species was a major factor in the Irish potato famine in the 1840s and to this day remains the most destructive oomycete pathogen in agriculture, causing over $6 billion worth of damage annually^1^. Current practices to control *P. infestans* rely on chemicals that lead to the development of resistance^3^, can have negative effects on the environment (due to runoff and persistence) and pose a threat to non-target organisms. Understanding the molecular mechanisms driving *P. infestans* infection is therefore key for developing targeted and sustainable approaches for crop protection.

The plant cell wall is the first barrier against pathogens and is composed of a complex network of cellulose microfibrils embedded in hemicellulose and lignin, plus a layer of pectin forming the bulk of the middle lamella between cells^4^. The plant cell wall architecture and composition have driven the evolution of a diverse range of carbohydrate active enzymes (CAZymes^5^) in both fungi and oomycetes^6,7^. These enzymes are actively secreted during infection and disrupt the plant cell wall, releasing oligosaccharides that are recognised as damage-associated molecular patterns (DAMPs) by host receptors, thereby triggering the plant innate immune response^8^. Among these DAMPs, oligogalacturonides (OGs) - pectin fragments released by polygalacturonase activity - are specifically sensed by plant cell wall-associated kinases, triggering a cascade of immune responses, including the rapid and transient production of hydrogen peroxide (H₂O₂) via NADPH oxidase activation^9^. This process is one of the earliest defence responses in plants and plays a critical role in both local and systemic immunity^10^.

Using *P. infestans* as a model system, we recently showed that phytopathogenic oomycetes secrete lytic polysaccharide monooxygenases (LPMOs) to oxidatively cleave the pectin backbone and drive plant infection^7^. This discovery prompted us to investigate the possible roles of other types of oxidoreductases in plant-oomycete interactions. Among genes that were induced over 10-fold and coding for predicted secreted proteins, we identified a putative catalase (*PITG_07143*), cellobiose dehydrogenase (*PITG_07303*) and several berberine bridge enzyme-like proteins (BBEs). In particular, the gene expression pattern and strong induction of three BBE genes (*PITG_02928*, *PITG_02930* and *PITG_02935*) suggested potential functional relevance during plant infection. BBEs are a diverse class of flavin adenine dinucleotide (FAD) dependent oxidases involved in the biosynthesis of alkaloids (plants, fungi), antibiotics (bacteria), steroids (fungi), as well as in the oxidation of mono- and oligosaccharides (plants, fungi)^11^. BBEs are classified within the auxiliary activity 7 (AA7) family in the CAZy database^5^, and are part of the vanillyl alcohol oxidase (VAO) superfamily featuring an N-terminal FAD binding domain and a C-terminal substrate binding domain. The type of coordination of the FAD cofactor can significantly influence the redox properties, stability, substrate specificity and catalytic mechanism of the enzyme. Most characterised AA7s harbour a bi-covalently tethered (through a cysteinyl and histidyl residue) FAD cofactor^11^. However, recent work has revealed the existence of fungal AA7s with mono histidyl-tethered FAD and oomycete AA7s with predicted mono cysteinyl-tethered FAD^12^, suggesting biological roles yet to be elucidated.

AA7s have previously been hypothesised to act as effectors in *Phytophthora*, with some shown to be secreted during hyphal growth in liquid media^13,14^. More recently, while this manuscript was under review, Turella et al.^15^ published the biochemical characterisation of AA7s produced by *Phytophthora sojae* during soybean infection, identifying them as OG oxidases and proposing that the resulting oxidised OGs may evade recognition by the plant immune system, potentially contributing to stealth infection. Their study provided valuable biochemical insights and raised compelling hypotheses about the role of AA7s in virulence.

Here, we extend these findings by presenting a multi-layered body of evidence that validates and expands the proposed immune-modulatory function of *Phytophthora* AA7s *in planta*. We show that *P. infestans* AA7 genes are strongly induced during infection and encode secreted proteins that specifically oxidise OGs at their reducing end, hampering their ability to induce a hydrogen peroxide burst in both *Arabidopsis* and tomato plants. Crucially, we also show that these AA7-oxidised OGs actively suppress ROS production normally triggered by native OGs, indicating an active role in antagonising ongoing defence responses in plants. Live-cell confocal imaging of fluorescently tagged AA7 in *P. infestans* reveals localisation at germ tube tips and haustoria during early infection, positioning these enzymes at key host-pathogen interfaces where OGs are released through degradation of the plant cell wall during penetration. Finally, gene silencing experiments followed by tests for pathogenicity in potato demonstrate a marked reduction in lesion size upon knockdown of AA7 genes, establishing their essential role in virulence.

Together, these findings reveal a novel microbial strategy for disarming plant defence signals and promoting infection, paving the way for developing innovative strategies to fight crop diseases and enhance global food security.

## Results

### AA7s are strongly induced in oomycetes during plant infection and present unique structural features

Originally, seven genes were annotated as BBEs in the genome of *P. infestans* strain T30-4^16^. However, we closely inspected RNA-seq data previously collected from sporangia and during infection of tomato leaves^7^ and found no support for the predicted introns in *PITG_06051* and *PITG_06585*. Instead, both gene sequences contain premature stop codons, leading to truncated open reading frames (ORFs) of 85 and 141 amino acid residues, respectively. This left five high confidence genes: *PITG_02928 (PiAA7A)*, *PITG_02930 (PiAA7B)*, *PITG_02935 (PiAA7C)*, *PITG_06591* (*PiAA7D*), and *PITG_20764 (PiAA7E)*. All the corresponding proteins carry predicted signal peptides for secretion and are recognised as Auxiliary Activity (AA) family 7 by the online annotation tool DBCAN3^17^, although none of them are currently classified within the AA7 family in the online CAZy database^5^.

**Fig. 1:**
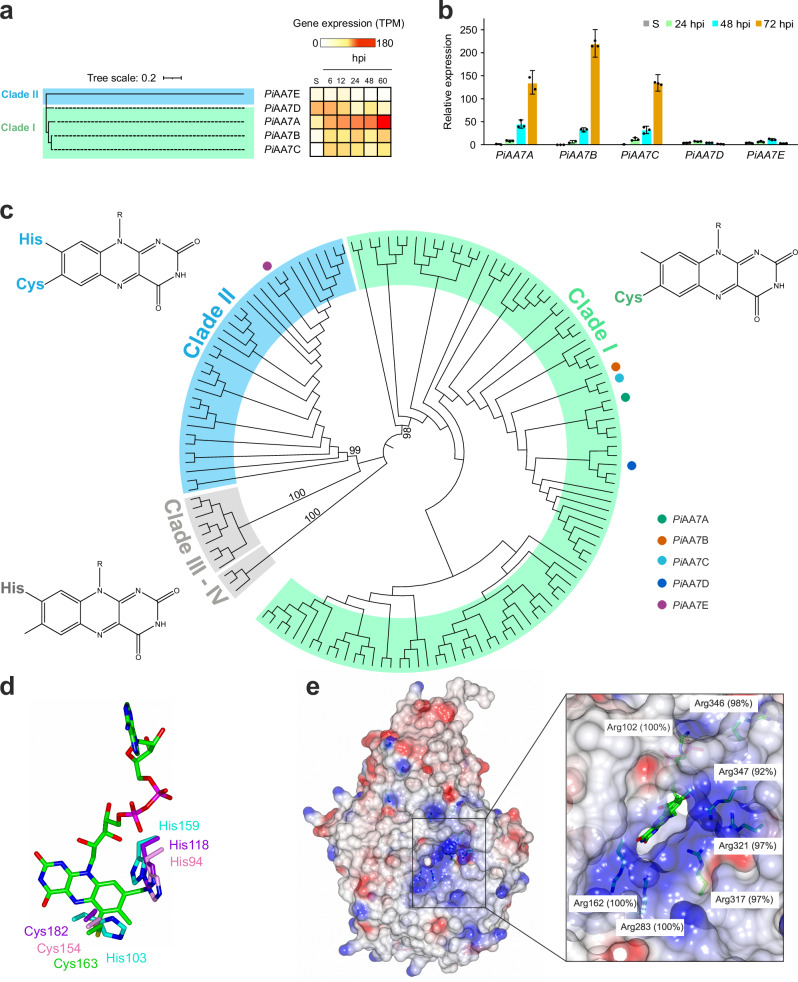
Gene expression, phylogeny and structural properties of AA7 enzymes in oomycetes. **a** Neighbour-joining phylogenetic tree of *Pi*AA7A-E proteins, and normalised gene expression of *PiAA7A-E* genes based on RNA-seq data of *P. infestans* infecting tomato leaves (6, 12, 24, 48 and 60 h post-inoculation, hpi). Data were obtained from ref. ^7^. S: sporangia. TPM: transcripts per kilobase million. **b** RT-qPCR of *PiAA7A-E* in *P. infestans* infecting potato leaves at 24, 48 and 72 hpi. Gene expression is shown relative to *PiAA7A* level in sporangia, which was assigned the value 1.0. S: sporangia. Error bars represent 95% confidence intervals calculated using three technical replicates (*n* = 3) for each sample within the RT-qPCR assay. Assays repeated on three independent occasions (*n* = 3), using leaf material from three independent infection time courses for RNA isolation and subsequent cDNA synthesis, yielded similar profiles of transcript accumulation (Supplementary Data 1). **c** Neighbour-joining phylogenetic tree of oomycete AA7s. Clades I, II and III-IV are highlighted in green, blue and grey, respectively. For each clade, the typology of predicted FAD tethering is also shown. The five AA7 isoforms from *P. infestans* are indicated using coloured circles. Bootstrap values are indicated by numbers. **d** FAD cofactor coordination in *Pi*AA7A (green, representing Clade I), UniProt sequence G4ZRJ9 from *P. sojae* (purple, Clade II), UniProt sequence A0A1V9ZHL9 from *Achlya hypogyna* (cyan, Clades III-IV) and chitooligosaccharide oxidase (ChitO, PDB file 6Y0R) from *Fusarium graminearum* (pink), following superimposition of all models relative to ChitO. The canonical cysteine residue involved in covalent binding to the isoalloxazine ring is conserved in Clade I and II, whereas the canonical histidine residue is present only in Clade II, III and IV. Models for all clades were created using AlphaFold3. See Methods for more details. **e** Electrostatic surface potential of *Pi*AA7A, with positively charged residues (in blue) marking the boundaries of the entrance of the active site. Conservation scores of these residues within Clade I were calculated using ConSurf^20^ and are indicated in white boxes. Source data are provided as a Source Data file.

In transcriptomic datasets we collected during previous studies of *P. infestans* infecting tomato plant leaves^7^, strong induction of *PiAA7A-C* was observed at 6, 12, 24, 48 and 60 hours post-inoculation (hpi), while *PiAA7D* was most expressed in sporangia and at 6–12 hpi (Fig. 1a). We complemented this with reverse transcription quantitative polymerase chain reaction (RT-qPCR) to investigate the expression profile of the five *PiAA7* genes during infection of potato leaves (Fig. 1b). Strong up-regulation was observed for established transcriptional markers associated with developing infection, including the *P. infestans* RxLR effector *PITG_04314*^18^ and *PITG_12808*^19^ (Supplementary Data 1). Genes *PiAA7A-C* exhibited the greatest transcript abundance and up-regulation during plant infection, whereas *PiAA7D* and *PiAA7E* mRNAs were less abundant and not significantly induced (Fig. 1b).

To identify conserved as well as novel features that may relate *P. infestans* AA7s to plant pathogenesis, we carried out a phylogeny of AA7s across oomycetes with different lifestyles and host preferences, covering strict plant pathogens (*Albuginales*, *Peronosporales*, *Pythiales*) and animal pathogens (*Saprolegniales*). In parallel, we performed residue conservation analysis using ConSurf^20^, used this information to inspect AlphaFold3 models across the phylogenetic tree and identified clade-specific residues and structural motifs. Full-length oomycete AA7s exhibit the canonical AA7 fold, consisting of an FAD-binding F domain and a substrate-binding S domain, with a central β-sheet flanked by α-helices. In our phylogenetic tree we identified four main clades based on conservation of key residues predicted to be involved in tethering the FAD cofactor, hence likely affecting redox potential, substrate binding and catalytic properties^21^ (Fig. 1c). Clade I features a cysteine involved in mono-covalent FAD binding (6-S-cysteinyl-FAD) and is expanded in plant pathogenic oomycetes, averaging five isoforms in *Phytophthora* species including all strongly induced *P. infestans* isoforms (*Pi*AA7A-C). In Clade II, FAD is tethered both through cysteine (6-S-cysteinyl) and histidine (8α-N1-histidylation) and typically averages one copy per species. Clade III and Clade IV are only found in *Saprolegniales* and present high conservation of the histidine for 8α-N1-histidylation, whereas the cysteine normally involved in 6-S-cysteinyl binding is not conserved and is substituted by histidine, asparagine, serine or alanine (Fig. 1d). While members of Clades I and II are predicted to be secreted and thus likely involved in oxidation of extracellular molecules, Clade III and Clade IV AA7s do not present a secretion peptide, suggesting roles in intracellular metabolism. Interestingly, oomycete AA7s from Clade I and II, but not III and IV, share an aromatic amino acid cluster putatively involved in catalysis, consisting of a catalytic base tyrosine and a tyrosine/phenylalanine (Y461 and F104 in *Pi*AA7A, respectively, Supplementary Fig. 1) likely involved in stabilising the catalytic base^11,12^.

The aromatic residue that normally stacks onto the neutral saccharide unit penultimate to the reducing end^12^ is conserved in Clade II but substituted with nonpolar aliphatic residues in Clades III and IV and a serine in Clade I (Supplementary Fig. 2), again suggesting distinct substrate specificity between clades. Analysis of sequence conservation and electrostatic surface potential of *Phytophthora* AA7 models reveals that only Clade I members feature a highly conserved patch of positively charged arginine residues surrounding the opening which leads to the solvent-exposed FAD cofactor, thus suggesting specific interactions with negatively charged substrates (Fig. 1e and Supplementary Fig. 3a). Interestingly, one of these positive surface residues (Arg102 in *Pi*AA7A) substitutes the highly conserved histidine involved in 8α-N1-histidylation of FAD in Clades II–IV (Supplementary Fig. 3b).

### *P. infestans* AA7s oxidise plant oligogalacturonides at the reducing end

*Pi*AA7s belonging to Clade I were identified as the most induced and expressed throughout infection and were therefore selected for further biochemical characterisation. The codon-optimised, polyhistidine-tagged *PiAA7A-D* sequences were expressed individually in *Pichia pastoris* and purified through nickel affinity and size exclusion chromatography (Supplementary Fig. 4a). Protein identity was confirmed for all four targets through mass spectrometry. Correct protein folding was recorded using thermal shift assays, with melting temperatures ranging between 50.9 and 60.8 °C across isoforms (Supplementary Fig. 4b). As expected^12^, ultraviolet-visible spectrophotometry (UV-vis) analysis revealed absorption peaks at ~385 and ~457 nm imparted by the FAD cofactor (Supplementary Fig. 4c).

**Fig. 2:**
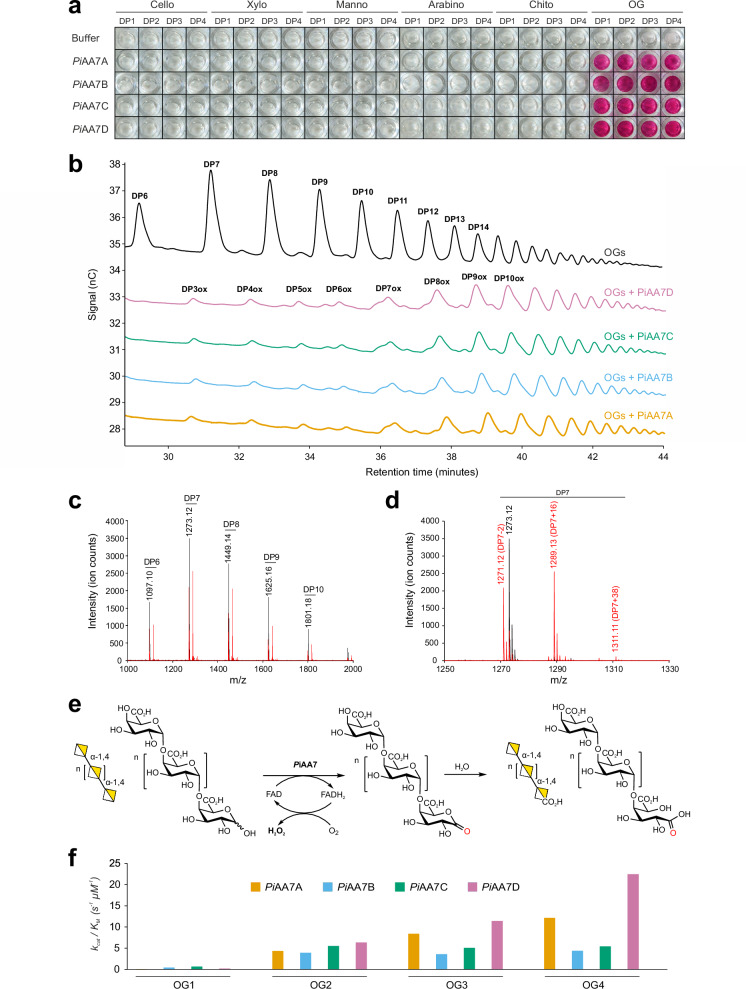
Biochemical characterisation of *P. infestans* AA7 enzymes. **a** Oxidase activity of *Pi*AA7s on commercial oligosaccharides (DP between 1 and 4, Megazyme) was detected through a colourimetric assay. Reactions were set up with 0.1 mM substrate, 20 nM enzyme, 0.1 mM AAP, 1 mM DCHBS and 2 U mL^−1^ HRP in 10 mM HEPES buffer plus 50 mM NaCl at pH 7, and incubated for 1 h at 20 °C. **b** HPAEC-PAD analysis of oxidised OGs. 150 nM *Pi*AA7A-D was incubated with 2 mg mL^–1^ long OGs (DP10-15, Elicityl) in 25 mM MES buffer pH 6 for 17 h at 20 °C. The identity of OGox peaks within the product ladder was determined based on retention times of OGox with DP2/3/4. **c** MALDI-TOF MS analysis of oxidised OGs. 600 nM *Pi*AA7A was incubated with 10 mg mL^−1^ long OGs (DP10-15, Elicityl) in 20 mM ammonium acetate buffer pH 6 for 12 h 20 °C. Substrate (black line) and products (red line) spectra are shown. **d** Expanded MALDI-TOF MS spectra for DP7 upon incubation with *Pi*AA7A. Native and oxidised species are labelled in black and red, respectively. *m*/*z* 1271.12: − 2 species, oxidised (C1-ketone, mono-sodiated). *m*/*z* 1273.12: native species (mono-sodiated). *m*/*z* 1289.13: + 16 species, oxidised (C1-aldonic acid, mono-sodiated). *m*/*z* 1311.11: + 38 species, oxidised (C1-aldonic acid, di-sodiated). **e** Schematic representation of OG oxidation by *P. infestans* AA7 enzymes. During substrate oxidation, two electrons and two protons are transferred to the FAD cofactor, reducing it to FADH_2_. FADH_2_ is oxidised by molecular oxygen (O_2_), regenerating FAD and producing hydrogen peroxide (H_2_O_2_). The OG is oxidised at the reducing end (C1, in red), resulting in a ketone which undergoes spontaneous hydration and conversion into an aldonic acid. The inserted oxygen atom is indicated in red. **f** Catalytic efficiency of *Pi*AA7A-D, expressed at *k*_*cat*_ */ K*_*M*_, across pure commercial OG standards with increasing DP (1–4). The original *k*_*cat*_ and *K*_*M*_ values are shown in Supplementary Data 2. Source data are provided as a Source Data file.

The pure recombinant enzymes were first qualitatively screened for activity against a panel of oligosaccharides with a degree of polymerisation (DP) from 1 to 4 - derived from cellulose, chitin, mannan, xylan, arabinan and polygalacturonic acid - by monitoring H_2_O_2_ production using a peroxidase-coupled assay. All four *Pi*AA7s showed specific and strong oxidase activity on short commercially supplied oligogalacturonides (OGs DP1/2/3/4), whereas no activity was detected on any of the other substrates tested (Fig. 2a). Subsequent time course quantitative colourimetric assays incubating *Pi*AA7s with short (DP1/2/3/4) and long OGs (average DP10-15) confirmed rapid oxidation of all tested substrates (Supplementary Fig. 5), which was validated through HPAEC-PAD (Fig. 2b). MALDI-TOF MS analysis of products released by *Pi*AA7s upon incubation with long OGs revealed disappearance of the native species and conversion into signature peaks (− 2, + 16 and + 38 species) compatible with oxidation at the reducing (C1) end^22^ (Fig. 2c, d and Supplementary Fig. 6a–f). Selective C1 oxidation of long OGs at the reducing terminus (Fig. 2e) was unambiguously confirmed by 1H-NMR analysis with characteristic loss of both H1 protons of an α/β anomeric mixture observed following enzyme-catalysed oxidation of an α-1,4-linked oligogalacturonide (Supplementary Fig. 7).

A pH of 6 was identified as optimal across isoforms and substrates (Supplementary Fig. 8) and used for detailed kinetic studies. All isoforms exhibited high turnover rates (*k*_*cat*_), with catalytic efficiency (*k*_*cat*_*/K*_*M*_) increasing for oligogalacturonides with higher degrees of polymerisation (Fig. 2f and Supplementary Data 2). For most enzyme and substrate combinations, the Michaelis-Menten model provided the best fit. However, a sigmoidal (allosteric) model was found to be more appropriate for *Pi*AA7A with OG3 and *Pi*AA7D with OG4, while a substrate inhibition model best described the kinetics of *Pi*AA7A with OG4 and long OGs, as well as *Pi*AA7D with long OGs (Supplementary Data 2). These surprisingly different kinetic behaviours suggest isoform-specific modes of substrate recognition, cooperative effects and substrate inhibition depending on both OG concentration and chain length.

### *Pi*AA7-oxidised OGs fail to trigger ROS signalling in plants

**Fig. 3:**
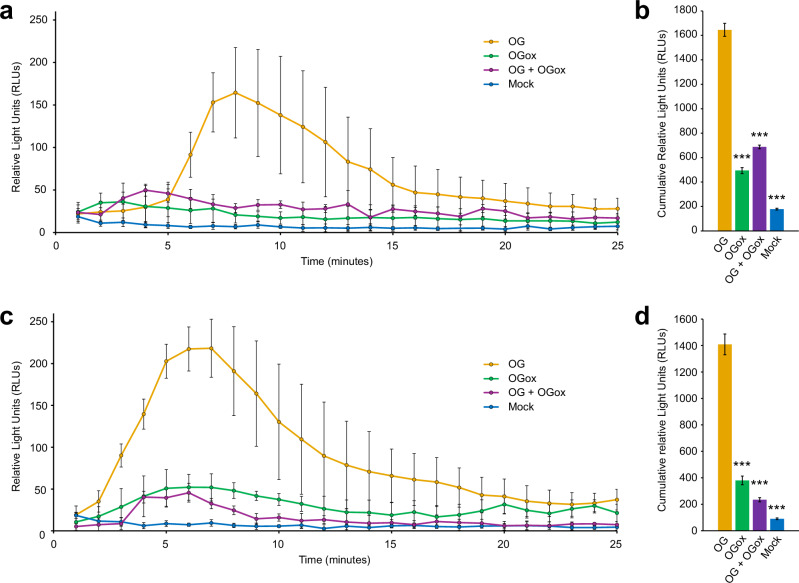
*In planta* oxidative burst detection following treatment with native and oxidised OGs. **a**, **b** Leaf discs of four- to five-week-old *Arabidopsis* were treated with: 0.2 mg mL^−1^ OG (long native OGs, DP10-15, Elicityl); 0.2 mg mL^−1^ OGox (long oxidised OGs DP10-15, following incubation with *Pi*AA7A); 0.4 mg mL^−1^ OG + OGox (long native and oxidised OGs, DP10-15, 0.2 mg mL^−1^ each); mock (buffer control). H_2_O_2_ production was monitored over time using a peroxidase-coupled luminescent assay based on luminol (see Methods). The values of quantification are displayed as an average of several replicates: 6 leaves (*n* = 6) for mock, 8 leaves (*n* = 8) for OGox, 12 leaves (*n* = 12) for OG and 5 leaves (*n* = 5) for OG + OGox. Only one disc was taken from each leaf, and only one leaf was taken from each individual plant. **c**, **d** Four- to five-week-old tomato plants cv. MicroTom were assessed under identical conditions to *Arabidopsis*. 6 leaves (*n* = 6) were used for treatments with OG, OGox, and mock, and 5 leaves (*n* = 5) for OG + OGox. Panels (**a** and **c**) show the time course experiments across 25 minutes. Panels (**b** and **d**) show the cumulative signal over 25 min. Error bars represent the propagated standard error of the mean. *P*-values were calculated using a two-tailed *t* test assuming unequal variances (Welch’s *t* test). ***: *P* < 0.001. P values in panel (**b**) are 0.00001324 (OG vs OGox), 0.00000011 (OG vs mock) and 0.00021120 (OG vs OG + OGox). *P*-values in panel (**d**) are 0.00002736 (OG vs OGox), 0.00000004 (OG vs mock) and 0.00000055 (OG vs OG + OGox). Source data are provided as a Source Data file.

To assess the impact of *Pi*AA7-oxidised OGs on plant immune signalling, we examined their effect on the production of ROS, a hallmark of the early response to DAMPs such as long OGs (DP10-15)^22–24^. We evaluated the short-term (0–25 min) H_2_O_2_ generation in leaf discs using a luminol-based assay, in response to different treatments. As expected, native long OGs induced a strong oxidative burst within the first 10 minutes in both *Arabidopsis* (Fig. 3a, b) and tomato (Fig. 3c, d). However, AA7-oxidised long OGs showed a much-reduced ability to induce this response in both plants. Moreover, co-treatment with native and oxidised OGs failed to trigger a full response, suggesting that the oxidised OGs interfere with the perception of, or downstream signalling from, native OGs.

### AA7s localise at the sporangia germ tube and haustoria during *P. infestans* infection

**Fig. 4:**
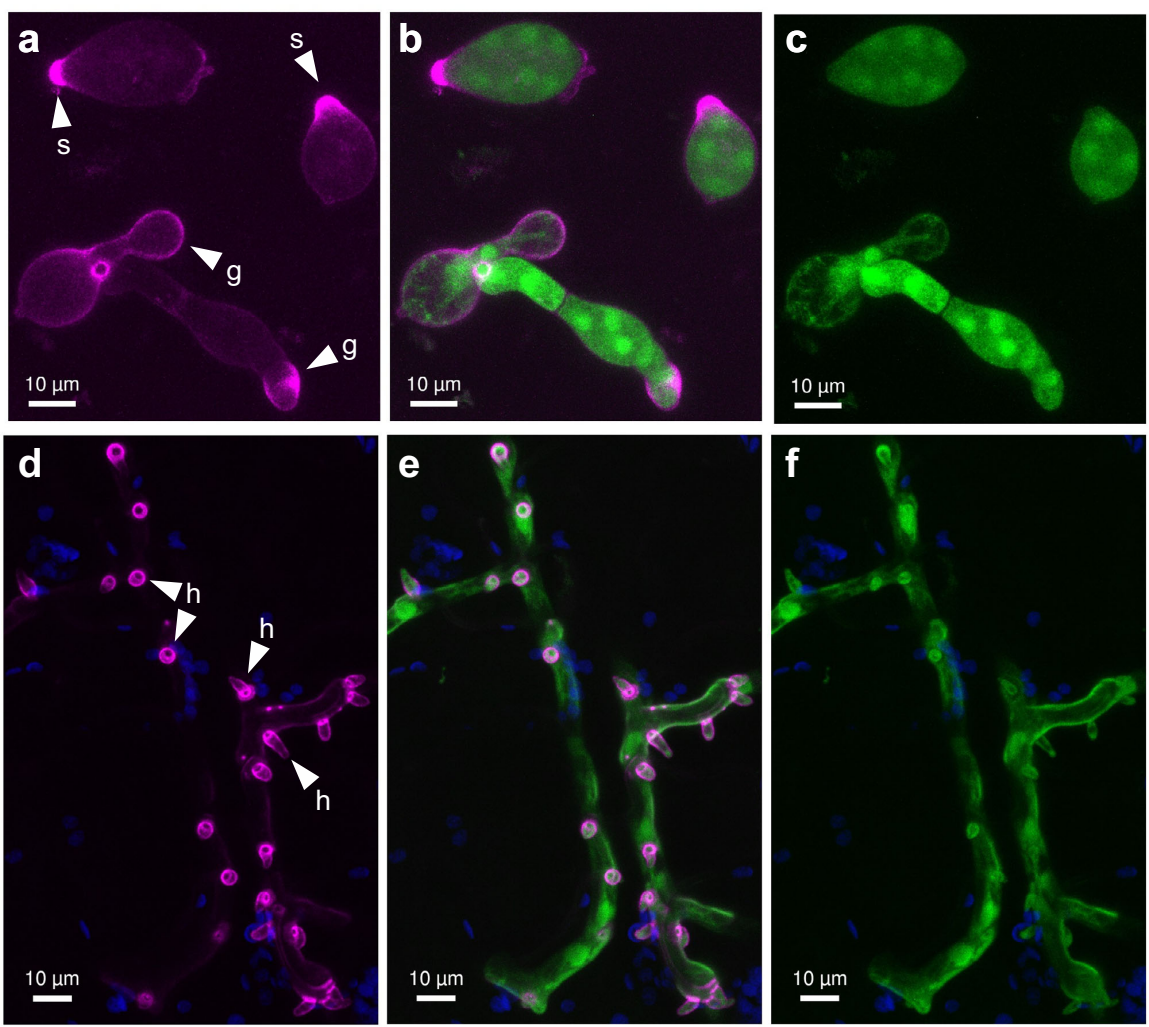
*Pi*AA7A localisation during infection of *Nicotiana benthamiana* leaves with *P. infestans*. Confocal projection images of *P. infestans* isolate 2006-3928A expressing C-terminal *Pi*AA7A-mScarlet fusion under the control of the *PiAA7A* native promoter and cytoplasmic mCitrine (green). *Pi*AA7A-mScarlet (pink) localised to tips of sporangia (s) and outlined germ tubes (g) on the surface of an *N. benthamiana* leaf (**a**–**c**). In infectious hyphae, mScarlet fluorescence outlined haustoria (h), especially the base of haustoria (**d**–**f**). Chlorophyll autofluorescence from the plant cells is shown in blue. Images (**a**–**c**) and (**d**–**f**) were collected at two- and four-days post-inoculation (dpi), respectively. mScarlet in pink (**a**, **d**), merged images with mScarlet in pink and mCitrine in green (**b**, **e**), cytoplasmic mCitrine in green (**c**, **f**). To aid interpretation, sporangial tips (s), germ tubes (g), and selected haustoria (h) are labelled in the images. Scale bars indicate 10 µm. Each image is representative of at least 10 images of independent infection points.

*Pi*AA7A was C-terminally tagged with monomeric Scarlet (mScarlet) fluorescent protein and expressed under the control of its native promoter in transgenic lines of *P. infestans* isolate 2006-3928A. The cytoplasm of these transformed lines was also labelled with untagged monomeric Citrine (mCitrine) fluorescent protein. Line 2928-mSc-mCit-5, which showed strong expression of both fluorescent markers and retained normal pathogenicity, was selected for most confocal imaging experiments. mScarlet tagged *PiAA7A* was shown to have the same transcription profile as the untagged *PiAA7A*, confirming that the promoter was functioning as expected (Supplementary Data 3). Since *PiAA7A* is upregulated from the start of *P. infestans* interaction with the host plant, with transcription rapidly increasing over the first three days of potato leaf infection (Fig. 1b), confocal microscopy was targeted to the leading edges of infection foci. Early after leaf inoculation, the *Pi*AA7A-mScarlet fusion protein expressed in transgenic *P. infestans* lines localised to the tips of sporangia (the papilla), outlining the germ tube as sporangia germinated, and especially the tip of the germ tube prior to host tissue penetration (Fig. 4a–c and Supplementary Fig. 9a–f). In the model solanaceous plant *Nicotiana benthamiana*, after leaf penetration, the *Pi*AA7A-mScarlet fusion protein was observed outlining parts of the infectious hyphae and the cell-penetrating projections called haustoria, particularly around the base of haustoria where the plant cell wall is breached (Fig. 4d–f and Supplementary Fig. 9g–l). The localisation of tagged *Pi*AA7A at haustoria was also observed during *P. infestans* infection of potato leaves (Supplementary Fig. 10). The secretion and extracellular localisation of *Pi*AA7A was confirmed through western blot analysis of the culture medium in which *P. infestans* constitutively expressing the *Pi*AA7A-mCherry fusion protein was grown (Supplementary Fig. 11).

### Silencing of *Pi*AA7 genes severely affects pathogen growth during plant infection

**Fig. 5:**
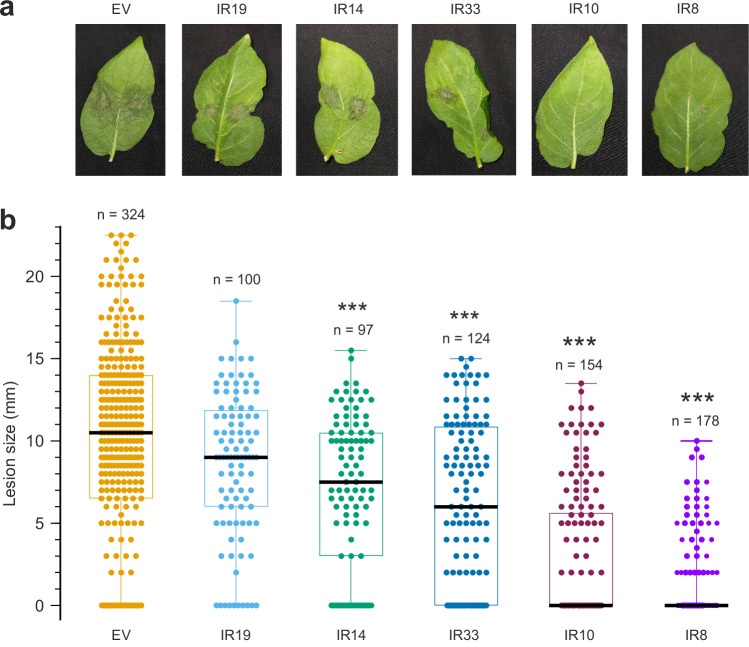
Effect of silencing *PiAA7A-D* genes on lesion size during infection of potato leaves 5 dpi. **a** Leaves were inoculated with *P. infestans* strain 2006-3928A stably transformed with a silencing plasmid targeting AA7s. Lines IR19, IR14, IR33, IR10 and IR8 were used. IR: Inverted Repeat (see Methods for more details). EV empty vector control. **b** Lesions were measured for all *P. infestans* lines. In each box plot, the black line shows the median, and box limits indicate the 25th and 75th percentiles. Whiskers extend from maxima (top) to minima (bottom). Each coloured circle represents the lesion area of an individual infection spot (see Methods for more details), and *n* is the number of lesions measured from each silenced line. *P* values were calculated using a two-tailed *t* test assuming unequal variances (Welch’s *t* test). ***: *P* < 0.0001. *P*-values in panel (**b**) are 0.086 (EV vs IR19), 0.00006802 (EV vs IR14), 0.00000001 (EV vs IR33), 0 (EV vs IR10) and 0 (EV vs IR 8). Source data are provided as a Source Data file.

We assessed the role of *P. infestans* AA7s in plant infection through transgene-mediated gene silencing. To simultaneously silence *PiAA7A-D*, a 508 bp fragment of *PiAA7A* largely conserved within Clade I was transformed into *P. infestans* as an inverted repeat (Supplementary Fig. 12). Five healthy geneticin-resistant *P. infestans* lines were isolated (Supplementary Fig. 13), with variable levels of AA7 gene silencing (Supplementary Data 4). Lines IR14, IR33, IR10 and IR8 exhibited a significant reduction in lesion size upon infection of potato leaves, compared to the empty vector control line (Fig. 5a, b). Furthermore, we observed a good correlation between the level of silencing of *PiAA7A-C* and lesion size across all silenced lines (Supplementary Fig. 14), indicating that isoforms A-C significantly contribute to virulence. Importantly, *PiAA7E* served as an internal specificity control for off-target silencing. This isoform is substantially divergent at the DNA level compared to *PiAA7A-D* and, unlike those isoforms, is not induced during infection (Fig. 1a, b). Consistent with this, we observed no reduction in *PiAA7E* expression in any of the silenced transformants, and its expression levels did not correlate with lesion size (Supplementary Fig. 14f). These observations strongly support the specificity of the silencing construct and reinforce the conclusion that the observed phenotypes are due to specific knockdown of the infection-induced AA7 isoforms.

## Discussion

In this study, we uncover a novel virulence strategy in oomycetes, in which FAD-dependent AA7 oxidases are secreted during infection and specifically oxidise the reducing end of OGs - key plant damage signals - to neutralise their elicitor activity, thereby evading host immunity and promoting infection. Our work reveals a new layer of complexity in host-pathogen interactions and expands the known biological functions of the AA7 family. This discovery positions AA7s alongside a growing arsenal of pathogen-secreted redox enzymes, such as peroxidases and catalases, that manipulate the host oxidative environment to suppress immune responses^25,26^.

Phylogenetic and gene expression analyses place these OG-active microbial AA7s within a distinct Clade I, which likely evolved from an ancestral Clade II member and uniquely expanded in the *Phytophthora* genus. Here, the coding genes are strongly induced during plant infection, mirroring the expression patterns of pectinolytic CAZyme families, including pectin methyl esterases (CE8), polygalacturonases (GH28), pectate lyases (PL3) and AA17 LPMOs reported in our previous work^7^, and more recently by Turella et al.^15^. This coordinated upregulation suggests a dual strategy: degradation of the pectin-rich middle lamella to penetrate the plant tissue and access nutrients, and simultaneous inactivation of immune-eliciting OGs to evade host defences. This strategy may not be restricted to oomycetes, as other phytopathogenic microbes - fungi and bacteria - have likely evolved analogous mechanisms to disable DAMP signals.

Our western blot and live-cell microscopy data show dynamic AA7 secretion and localisation patterns that align with critical stages of host infection, supporting a key role for *Pi*AA7s in *Phytophthora* pathogenicity. The *Pi*AA7-mScarlet fusion protein is clearly detected at the tips of sporangia and along the germ tube during germination, indicating that these enzymes are deployed from the onset of the interaction with the host plant. As infection progresses, the secreted protein accumulates at haustoria, strategically positioned to rapidly oxidise - and inactivate - these powerful DAMPs before they can trigger host defences. Haustoria, formed by fungi, oomycetes and parasitic plants, develop through localised plant cell wall degradation and invagination of the host plasma membrane, and are crucial for nutrient uptake and effector delivery during the biotrophic phase of the interaction^27,28^. Our findings place AA7 enzymes at the heart of this interface, suggesting a novel virulence strategy that manipulates host glycan signalling. Moreover, *Pi*AA7s exhibit robust catalytic activity across a broad pH range centred around pH 6, compatible with the mildly acidic environment of the plant apoplast^29^. Consistent with our findings, previous work identified two *P. sojae* AA7 proteins in the apoplastic fluid of infected soybean leaves using mass spectrometry analysis^30^. Interestingly, endogenous OG-specific AA7s produced by plants are also apoplastic^31^, suggesting that both host and pathogen have evolved to oxidise the immune-active glycans in the same extracellular compartment.

OG-active oomycete AA7s feature distinctive structural signatures that correlate with their unusual substrate specificity. These include a mono-cysteinyl tethered FAD, rather than the typical bi-covalent cysteine/histidine coordination found in most AA7 enzymes, and a conserved cluster of solvent-exposed arginine residues that confer a highly positive charge around the active site. These features were recently confirmed in the X-ray structures of *P. sojae* AA7s^15^, and likely facilitate electrostatic interactions with the negatively charged carboxylates of OGs, as previously hypothesised by Benedetti et al.^22^ for a small group of *Arabidopsis* AA7s (called “OGOX” proteins) also capable of oxidising OGs. Indeed, we find striking parallels between *Phytophthora* AA7s and *Arabidopsis* OGOX enzymes, as both groups exhibit similar positive charge distributions, substrate preference toward long OGs (DP ≥ 4), and specific oxidation at the reducing end of these molecules. Consistent with our *in planta* observations using *Pi*AA7-oxidised OGs, *Arabidopsis* OGOX oxidation products have also been shown to trigger a much-reduced oxidative burst while failing to induce expression of defence-related marker genes^22^. Phylogenetic analysis and surface charge distribution of predicted 3D protein models from *Solanaceae* strongly suggest that OG-active AA7s are also produced by natural hosts of *P. infestans*, including tomato and potato (Supplementary Fig. 15). The widespread occurrence of these OG-specific enzymes across diverse plant lineages points to a conserved, ancestral role in modulating OG-mediated signalling as part of the plant’s own regulatory machinery.

Together, our findings point to a remarkable case of convergent evolution. While plant OGOX enzymes are believed to modulate OG signalling during cell wall remodelling to prevent hyper-immunity^22,24^, our results suggest that *Phytophthora* species have independently evolved a molecular mimicry strategy to exploit the same chemical logic - using OG oxidation to escape immune detection during host invasion. Supporting this hypothesis, galacturonic acid and its polymers - such as OGs - are absent from oomycetes and the Stramenopile lineage, including *P. infestans*^32^, indicating that the pathogen’s AA7 enzymes target exclusively plant-derived pectin fragments, not endogenous ones. This example of biochemical convergence aligns with a broader pattern in host-pathogen co-evolution, where pathogens evolve to mimic host molecules or proteins to subvert defences and promote infection^33,34^. This independent, yet convergent evolution is supported by broader phylogenetic analysis (Supplementary Fig. 16) and by functional evidence that silencing *PiAA7*s in *P. infestans* leads to a significant reduction in pathogenicity.

In our study, we used different plant species for specific experimental approaches - tomato for RNA-seq profiling, potato for RT-qPCR as well as gene silencing and pathogenicity assays, *Nicotiana benthamiana* and potato for confocal microscopy, *Arabidopsis* and tomato for ROS assays. It is worth noting that, aside from *Arabidopsis*, all plants used here belong to the *Solanaceae* family, and include the two most economically important hosts of *P. infestans*. This diversity of plant species across experiments supports the conclusion that *Phytophthora* AA7 enzymes suppress OG-triggered immunity via a broadly conserved mechanism, effective across diverse dicot plants. This aligns with our view that these enzymes serve as general-purpose virulence factors, allowing *Phytophthora* species to tap into a common, ancestral DAMP-based immune signalling system in plants - one that relies on OGs as conserved elicitors of defence.

In summary, our work uncovers a previously unrecognised microbial strategy to suppress plant immunity, identifying AA7 OG oxidases as key players in the molecular arms race between pathogens and plants. These insights offer exciting new opportunities for the development of innovative crop protection strategies targeting microbial pathogens.

## Methods

### Reagents

2,5-Dihydroxy benzoic acid (DHB), Trizma-Base (TRIS), 4-(2-hydroxyethyl)-1-piperazineethanesulfonic acid (HEPES), 2-(N-morpholino)ethanesulfonic acid (MES), NaOH, KOH 37%, HCl solution, horseradish peroxidase (HRP), 4-aminoantipyrine (AAP), 3,5-dichloro-2-hydroxy-benzensulfonic acid (DCHBS), hydrogen peroxide, KCl, K_2_HPO_4_, KH_2_PO_4_, KNO_3_, MgSO_4_, CaCl_2_, PEG3350, sucrose, glucose, sorbitol, mannitol and geneticin (G418) were purchased from Sigma, Melford or Fisher Chemicals. High-purity oligosaccharides with DP between 1 and 4 were purchased from Megazyme or Sigma. Long oligogalacturonides (DP10-15, product code GAT114) were purchased from Elicityl.

### Bioinformatics

The sequences for the *P. infestans* AA7s *PITG_02928* (NCBI GenBank accession XM_002907740), *PITG_02930* (NCBI Genbank accession XM_002907741), *PITG_02935* (NCBI Genbank accession XM_002907745), *PITG_06585* (NCBI Genbank accession XM_002998628), *PITG_06591* (NCBI Genbank accession XM_002998633) and *PITG_20764* (NCBI Genbank accession XM_002895280) were obtained from FungiDB (https://fungidb.org/fungidb/app). The gene models were verified using previous RNAseq datasets mapped to the *P. infestans* genome sequence^7^ and inspected using Tablet^35^. 3D models of *P. infestans* AA7s were obtained using the online AlphaFold3 server https://alphafoldserver.com/, including FAD as ligand, and were superimposed using CCP4MG^36^. The NCBI predicted protein for *PITG_20764* (*Pi*AA7E) was found to miss a small stretch of amino acid residues near the N-terminus, which was highly conserved in the orthologues from other *Phytophthora* species and included the conserved histidine involved in covalent binding to the FAD cofactor. *Pi*AA7E was therefore excluded from structural analysis, and its orthologue from *P. sojae* (Uniprot ID G4ZRJ9, 85% sequence identity) was used as a representative of Clade II in Fig. 1d and Supplementary Figs. 1–3.

Phylogenetic analyses were carried out as follows. PFAM family PF08031 (“Berberine and berberine like”) was accessed in InterPro (version 103.0, updated 28 Nov 2024), and all 209 oomycete sequences were selected and downloaded. To carry out a wider phylogeny, we also downloaded from InterPro 27 protein sequences from *Arabidopsis*, 28 from *Solanum tuberosum*, 17 from *Solanum lycopersicum*, plus representative plant pathogenic Ascomycota (18 from *Gibberella zeae* and 20 from *Pyricularia oryzae*) and Basidiomycota (18 from *Puccinia graminis* and 23 from *Rhizoctonia solani*). Only sequences between 400 and 650 residues were kept and aligned using T-Coffee^37^. The multiple sequence alignment files were opened in Mega11 and used to generate neighbour-joining trees. The phylogenetic trees were visualised using iTOL (https://itol.embl.de/) and edited with the graphic tool CorelDraw Graphics Suite 2023.

For ConSurf analysis of Clade I AA7s, all 95 sequences belonging to Clade I in our phylogenetic tree were aligned using T-Coffee^37^, and the alignment was uploaded to the ConSurf server for analysis^20^ using standard parameters.

### *P. infestans* culture conditions

*P. infestans* isolate 2006_3928A was maintained at 18 °C in darkness on rye-sucrose agar^38^ amended with ampicillin (50 µg mL^−1^) and pimaricin (0.001% w/v). Transgenic strains of *P. infestans* were maintained on the same medium, with the addition of geneticin (G418; 10 µg mL^−1^). Comparison of growth rates of *P. infestans* lines was carried out as follows. 15 mm diameter circular plugs from growing cultures were transferred to fresh rye agar plates with no selective antibiotic. Cultures were incubated at 18 °C in the dark, and colony diameter was measured over time.

### Plant growth conditions for *P. infestans* infections

Potato cultivar Maris Piper and *N. benthamiana* plants were grown in a glasshouse under a 16 h day at 20−22 °C and an 8 h night at 18 °C. Supplementary lighting was automatically provided when the ambient light dropped below 200 W m^−2^.

### Leaf inoculation with *P. infestans*

Leaflets were collected from pre-flowering potato plants (cv. Maris Piper) and placed abaxial side facing upwards on moistened paper in clear plastic boxes. Alternatively, for confocal microscopy of *N. benthamiana*, the first fully expanded leaves were taken from six-week-old plants.

Sporangia were harvested by flooding 14-day old rye-sucrose agar cultures, rubbing with a spreader to dislodge sporangia, filtering through 70 µm nylon mesh, and centrifugation at 1000 × *g* for 5 min. Sporangia droplets (10 µl, 1 × 10^5 ^mL^−1^) were pipetted either side of the midrib on the abaxial side of the leaves placed on moistened paper in clear plastic boxes. Inoculated leaves were incubated in sealed plastic boxes at 20 °C in darkness for 18 h, then alternating 16 hours light / 8 h dark for up to six days.

### RT-qPCR of AA7 sequences in *P. infestans* isolate 2006-3928A during infection of potato leaves

Leaves of potato cv. Maris Piper were drop-inoculated with sporangia (1 × 10^5 ^mL^−1^) of *P. infestans* isolate 2006-3928A. Each of the 3 boxes of potato leaves was inoculated with an independently prepared batch of sporangia. Samples were collected from multiple leaves within each box at 24, 48, and 72 h post inoculation by taking leaf discs around inoculation sites. Samples were snap frozen in liquid nitrogen and stored at −70 °C until used for RNA isolation. Total RNA was extracted using the Nucleospin RNA Plant kit (Macherey-Nagel), omitting the on-column DNase digestion. TURBO DNase (Thermo Fisher) was used to digest genomic DNA. First-strand cDNA was synthesised from 20 μg total RNA using the Maxima First Strand cDNA synthesis kit, following manufacturers’ instructions (Thermo Fisher). Reactions and thermal cycling for RT-qPCR were as described by Wang et al.^27^ in a StepOne Real-Time PCR System (Applied Biosystems); primer sequences used in the reactions are listed in Supplementary Data 1. Calculation of relative gene expression used the ΔΔCt method^39^. For biological replicate 1 (Rep 1), the geometric mean of three endogenous control genes - ActinA (PITG_15117; EEY63399), casein kinase II β subunit (PITG_02745; EEY64204), and galactose oxidase (PITG_09862; EEY56344) - was used. Replicates 2 and 3 were normalised to actin expression alone, due to the AA7 transcripts being frequently undetectable in sporangia samples (Supplementary Data 1). Relative expression of all *P. infestans* AA7s was normalised against expression levels in *Pi*AA7A sporangia, as described in Grenville-Briggs et al.^40^. As the infection progresses at different speeds at independent infection spots initiated with the same inoculum on the same leaf, we used a mixture of material from many infection spots on several leaves for RNA extraction for each RT-qPCR sample and technical replicates within each experiment. Assays repeated on three independent occasions, using leaf material from three independent infection time courses for RNA isolation and subsequent cDNA synthesis, generated similar expression profiles (Supplementary Data 1). The same approach was taken to evaluate the PITG_02928-mScarlet native promoter using primers targeting native and transgene expression.

### DNA cloning for fluorescent tagging of *Pi*AA7A and gene silencing of AA7s in *P. infestans*

For C-terminal tagging with mCherry for constitutive expression, the *Pi*AA7A coding sequence was cloned into pmCitrine:mCherry^41^. For C-terminal tagging of *Pi*AA7A with mScarlet fluorescent protein, the pDUAL-mScarlet vector was generated by replacing the *Bremia lactucae* Ham34 promoter and mCherry in the pmCitrine:mCherry vector with promoterless mScarlet. Full-length *PiAA7A* with a 601 bp fragment preceding the start codon was PCR amplified (primers in Supplementary Data 1), digested with *AgeI* and *SbfI* restriction endonucleases (New England Biolabs), and ligated into pDUAL-mScarlet, also digested with these enzymes. Ligated plasmids were electroporated into *Escherichia coli* DH10B cells.

The pmCit-N-PcIR silencing vector was generated by replacing the mCherry cassette in the pmCitrine:mCherry vector with the inverted repeat cassette containing the *P. capsici* 40S ribosomal protein promotor^42^ followed by 3 cloning sites for a sense orientation fragment, a 71 bp intron from the *P. infestans* ste20 gene^43^, 3 cloning sites for an antisense orientation fragment, and a *P. capsici* 40S ribosomal protein gene terminator.

In an attempt to simultaneously silence multiple Clade I members of the AA7 gene family through generation of inverted repeats for initiation of gene silencing, a 508 bp fragment of *PiAA7A* representing a conserved part of the DNA sequence (Supplementary Fig. 12) was PCR amplified from genomic DNA of P*. infestans* isolate 2006_3928A with Phusion^TM^ polymerase using reaction conditions recommended by the manufacturer (New England Biolabs). The sense orientation fragment was digested with *PacI* and *MluI* restriction endonucleases, ligated into the corresponding sites of the silencing vector, and electroporated into *E. coli* DH10B cells. One of the positive clones was then used to clone the antisense fragment of *PiAA7A* into the *SbfI* and *AgeI* restriction endonuclease sites to yield the inverted repeat construct. All constructs were verified by sequencing.

### *P. infestans* transformation and gene silencing

*P. infestans* was transformed using a polyethylene glycol-CaCl_2_-lipofectin protocol^38^. Transgenic *P. infestans* were selected on rye-sucrose agar containing 5 µg mL^−1^ geneticin, then transferred to the same medium containing 10 µg mL^−1^ geneticin.

Recovered transformed lines were transferred to and maintained on rye-sucrose agar amended with 10 µg mL^−1^ geneticin antibiotic. Spot inoculation (10 µl, 1 × 10^5 ^mL^−1^) of silenced lines - referred to as “Inverted Repeat”, IR lines - on potato leaves (cv. Maris Piper) was used to assess disease development and measure gene expression during infection. Disease development was assessed as lesion diameter (mm) measured at 5 dpi. Samples for RT-qPCR were collected at 2 dpi for each line using leaf discs around inoculation sites. RNA extraction, cDNA synthesis and RT-qPCR were performed as described earlier, with the exception that only *ActinA* was used as an endogenous control. Recovered *Pi*AA7A-fluorescent protein fusion transformants were screened by confocal microscopy and western blot.

### Confocal microscopy

*N. benthamiana* and *S. tuberosum* leaf pieces inoculated with *P. infestans* expressing *Pi*AA7A-mScarlet were imaged using a Nikon A1R confocal microscope with a 40 × water immersion lens. Red fluorescence from mScarlet was imaged using 561 nm excitation, and emissions were collected between 570 and 620 nm. Fluorescence from mCitrine was imaged with 514 nm excitation, and emissions were collected between 525 and 555 nm. Chlorophyll autofluorescence was collected between 663 and 738 nm. The pinhole was set to 1.2 Airy units for the longest wavelength fluorophore. Image processing for figures was conducted using OMERO, or Nikon NIS-elements AR 4.30.00 and Adobe Photoshop.

### Secretion assay, SDS-PAGE and western blot

Secretion of *Pi*AA7A from *P. infestans* mycelia was confirmed using protein samples from a constitutively expressed *Pi*AA7A-mCherry fusion. Briefly, 4-day old mycelia were incubated in 1 mL lima bean media for 24 h. Mycelia were removed, dried, and resuspended in 100 µL 2 × SDS loading buffer. The remaining culture filtrate was centrifuged at 1000 × *g* for 10 min to remove debris, precipitated with chloroform/methanol and resuspended in 60 µL 2 × SDS loading buffer. Protein samples were heated to 95 °C for 10 min, centrifuged at 13000 × *g* for 10 min, and the supernatant taken for SDS-PAGE.

Samples were loaded onto 4–12% Bis-Tris NuPAGE gels (Invitrogen) run with 1 × MES buffer at 200 V for 40 min. Gels were blotted onto a nitrocellulose membrane for 1 h at 30 V. Membranes were blocked with 2% fish gelatine (Merck) in 1 × PBS. Primary antibodies for mouse α-RFP 6G6 (ProteinTech) and rabbit α-histone H3 (Abcam) were added at 1:3000. The membrane was washed in PBS-T before addition of the secondary antibody; Goat anti-mouse IRdye 800CW and Goat anti-rabbit IR dye 680RD (LICORbio) at 1:15000 dilution. Membranes were washed before imaging on the LICOR Odyssey system. Fast Green total protein stain (LICORbio) was used according to the manufacturer’s instructions before taking another image on the LICOR Odyssey.

### Cloning and heterologous expression of AA7 sequences from *P. infestans*

Codon optimised sequences of *Pi*AA7A-D, including their native signal peptides, were cloned into the pPINK^TM^ vector in frame with a short linker followed by a C-terminal 6his-tag (GSGSGSHHHHHH). Plasmids were linearised using *SpeI* and electroporated into PichiaPink^TM^ competent cells. White transformants were used to inoculate 5 mL Buffered Glycerol-complex Media (BMGY; 0.1 M potassium phosphate buffer pH 6, 1% yeast extract, 2% peptone, 1.34% yeast nitrogen base, 4 × 10^−5^% biotin, 1% casamino acids, 1% glycerol) and grown for 24 hours, 220 rpm, 30 °C; the following day, 2 × 1 mL of this was used to inoculate 2 × 150 mL BMGY cultures in 500 mL baffled flasks and grown for 20 h, shaken at 180 rpm, 30 °C. The 300 ml starter culture was directly inoculated into the conditioned fermentation vessel, and the culture was then grown until all the glycerol had been consumed (determined by dO_2_ spike). The cell biomass was further increased by a fed-batch phase on 50% (w/v) glycerol containing 12 mL L^−1^ of filter-sterilised PTM_1_ trace salts, at a feed rate of 18 mL h^−1^ L^−1^ of initial fermentation volume for 4 h. To induce expression of enzyme targets, a 100% methanol feed with PTM_1_ salts (12 mL L^−1^) was then added at 2 mL h^−1^ L^−1^ of initial fermentation volume for 4 h. When the culture had adapted to the methanol feed rate, had a steady DO % and had a fast DO spike after stopping the methanol source, the methanol feed rate was increased to 4 mL h^−1^ L^−1^ of initial fermentation volume for 16 h. The remaining cultivation was performed at the increased feed rate of 6 mL h^−1^ L^−1^ of initial fermentation volume. After 96 hours, the culture was harvested and centrifuged at 7500 × *g* for 20 min to remove the cells. The supernatant was decanted and stored at − 70°C.

### Protein purification

*Pichia* cultures containing soluble protein (72 to 96 h fermentation) were centrifuged at 30,200 × *g* for 20 min at 4 °C (Sorvall Lynx 6000) to pellet cells. Supernatants were adjusted to pH 6.8 using potassium hydroxide before purification via affinity and size exclusion chromatography. Cleared supernatants were applied to a HisTrapTM FF crude 5 mL column using an ÄKTA Start FPLC (Cytiva), using 0.02 M NaPO_4_ pH 7.4, 0.5 M NaCl for binding, and 0.02 M imidazole for washing in the same buffer (5 CV), and 0.5 M imidazole in the same buffer for elution (5 CV). Peak fractions containing the target protein were selected based on absorbance at 280 nm and checked by Coomassie-stained (InstantBlue, Abcam) SDS-PAGE gels, then pooled and concentrated if necessary (PALL 10 kDa MWCO spin columns) before applying to a Superdex 75 or 200, 26/600 column (Cytiva) equilibrated with 0.02 M HEPES pH 7 plus 0.1 M NaCl. Eluted peak fractions were analysed by SDS-PAGE and Coomassie staining before pooling.

### Protein ID by mass spectrometry

In-gel tryptic digestion of proteins separated by SDS-PAGE and stained with InstantBlue Coomassie protein stain (Abcam) was performed after reduction with dithioerythritol and S-carbamidomethylation with iodoacetamide. Gel pieces were washed two times with aqueous 50% (v/v) acetonitrile containing 25 mM ammonium bicarbonate, then once with acetonitrile and dried in a vacuum concentrator for 20 min. Sequencing-grade, modified porcine trypsin (Promega) was dissolved in 50 mM acetic acid, then diluted 5-fold with 25 mM ammonium bicarbonate to give a final trypsin concentration of 0.02 µg µL^−1^. Gel pieces were rehydrated by adding 25 µL of trypsin solution, and after 10 min enough 25 mM ammonium bicarbonate solution was added to cover the gel pieces. Digests were incubated overnight at 37 °C before extraction of peptides by washing three times with aqueous 50% (v/v) acetonitrile containing 0.1% (v/v) trifluoroacetic acid, and drying in a vacuum concentrator and reconstituting in aqueous 0.1% (v/v) trifluoroacetic acid. A 1 µL aliquot of each peptide mixture was applied directly to the ground steel MALDI target plate, followed immediately by an equal volume of a freshly-prepared 5 mg mL^−1^ solution of 4-hydroxy-α-cyano-cinnamic acid (Sigma) in 50% aqueous (v/v) acetonitrile containing 0.1% trifluoroacetic acid (v/v). Positive-ion MALDI mass spectra were obtained using a Bruker ultrafleXtreme in reflectron mode, equipped with a Nd:YAG smart beam laser. MS spectra were acquired over a mass range of *m/z* 800–4000. Final mass spectra were externally calibrated against an adjacent spot containing 6 peptides of known mass. For each spot, the ten most intense ions, with S/N greater than 8, were selected for fragmentation, which was performed in LIFT mode without the introduction of a collision gas. The default calibration was used for MS/MS spectra, which were baseline-subtracted and smoothed (Savitsky-Golay, width 0.15 *m/z*, cycles 4). Bruker flexAnalysis software (version 3.4) was used for spectral processing and peak list generation. Monoisotopic masses were obtained using a SNAP averaging algorithm (C 4.9384, N 1.3577, O 1.4773, S 0.0417, H 7.7583) and an S/N threshold of 2 for MS and 6 for MS2. Tandem mass spectral data searched against the cloned sequences using a locally-running copy of the Mascot programme (Matrix Science Ltd., version 3.0), through the Mascot Daemon interface (version 2.8). Search criteria specified: Enzyme, Trypsin; Fixed modifications, Carbamidomethyl (C); Variable modifications, Oxidation (M); Peptide tolerance, 100 ppm; MS/MS tolerance, 0.5 Da; Instrument, MALDI-TOF-TOF. Peptide matches were filtered to require an expected score of 0.05 or lower. Each protein sample returned nine peptides matching the provided sequence and hence confirmed the presence of the respective recombinant protein.

### Thermal shift analysis and protein concentration

The Thermofluor assay was conducted on the purified proteins using SYPRO^®^ Orange Protein Gel Stain (Life Technologies) in an Mx3005P qPCR System (Agilent Technologies). The intensity of the fluorescence was measured at a temperature gradient of 25–95 °C and converted into a melting curve (fluorescence changes against temperature) to determine the melting temperature (*T*_m_). Protein concentration was determined either with Bradford assay or from absorbance at 280 nm with a NanoDrop spectrophotometer (using molecular weight and extinction coefficient for the mature, 6his-tagged protein).

### UV-vis spectroscopy

Purified *Pi*AA7s were concentrated using 0.5 mL Amicon Ultra Centrifugal Filters with 3 kDa cut-off to 15–20 mg mL^−1^. UV-vis scans were performed on a Nanodrop ND-8000 scanning in the 200–800 nm region.

### In vitro activity assays

All reactions with purified *Pi*AA7s were performed at 20 °C, using different concentrations of enzyme and substrate, depending on the type of assay.

For the initial qualitative activity screen in 96-well format using different commercial oligosaccharides (DP1 to 4) as substrates, duplicate reactions were carried out in 10 mM HEPES plus 50 mM NaCl, pH 7, using 0.1 mM substrate and 20 nM enzyme for 1 h.

For pH optima, reactions were set up in 25 mM of either SHAM buffer (25 mM each of sodium acetate (NaOAc), HEPES, acetic acid and MES) at pH 5-8, or 25 mM Tris-HCl buffer at pH 9, using 0.05 mM OG1/2/3/4 and 10 nM enzyme, measuring absorbance at 514 nm on a BMG SpectrostarNano microplate reader. For pH optima using long OGs (DP10-15, Elicityl), reactions were carried out using 0.2 mg mL^−1^ substrate and 5 nM enzyme. All experiments were run in triplicate.

For kinetics, reactions were performed in 25 mM MES buffer pH 6 (which was found to be optimal for all four *Pi*AA7 isoforms) with 1 nM enzyme and the following ranges of substrate concentrations: OG1 - 0, 20, 40, 80, 160, 320, 640 and 1280 μM; OG2/3/4 - 0, 2.5, 5, 10, 20, 40, 80 and 100 μM; OG DP10-15 - 0, 6.25, 12.5, 25, 50, 100, 200 and 300 μg mL^−1^. Absorbance was measured at 492 nm using a Tecan Sunrise microplate reader for 31 cycles of 20 s each. All experiments were run in triplicate.

For HAPEC-PAD analysis of native and oxidised long OGs, 150 nM *Pi*AA7A-D was incubated with 2 mg mL^−1^ long OGs (DP10-15, Elicityl) in 25 mM MES pH 6 buffer for 17 h at 20 °C. Control reactions contained all components except the enzyme.

For MALDI-TOF MS analysis, 600 nM *Pi*AA7A-D was incubated with 10 mg mL^−1^ long OGs (DP10-15, Elicityl) in 20 mM ammonium acetate buffer pH 6 for 12 h at 20 °C.

All colourimetric, peroxidase-coupled assays (initial screen, pH optima, kinetics) were carried out in the presence of 0.1 mM AAP, 1 mM DCHBS and 2 U mL^-1^ HRP. Peroxidase assay data were always normalised to no enzyme controls, converting absorbance to μM H_2_O_2_ using a standard curve generated with known amounts of commercial H_2_O_2_, then calculating mean and standard deviation. Kinetic data were produced with Prism version 10.4.1 (GraphPad) built-in non-linear regression analyses with Michaelis-Menten, substrate inhibition or allosteric/sigmoidal models. Diagnostic tools were used to calculate goodness-of-fit and asymmetrical confidence levels at 95% for the data, and to compare the fit of models to find the one that best represents the data.

### Product analysis by mass spectrometry

One microliter of supernatant was mixed with an equal volume of 20 mg mL^−1^ 2,4,6-trihydroxyacetophenone (THAP) in 50% acetonitrile, 0.1% TFA on a SCOUT-MTP 384 target plate (Bruker). The spotted samples were then air dried before and analysed by mass spectrometry on an UltrafleXtreme matrix-assisted laser desorption ionisation-time of flight/time of flight (MALDI/TOF-TOF) instrument (Bruker) in positive mode, as previously reported^44^.

### Product analysis by HPAEC-PAD

OGs were analysed from undiluted samples via HPAEC using an ICS-6000 PAD system with an electrochemical gold electrode, a CarboPac PA1 2 × 250 mm analytical column and a CarboPac PA1 2 × 50 mm guard column (Dionex). Sample aliquots of 5 μL were injected and separated at a constant temperature of 30 °C. After equilibration of the column with 90% 0.1 M NaOH and 10% 0.1 M NaOH/1 M NaOAc for 2 min at a flow rate of 0.25 mL min^−1^, a 10-min linear gradient was started from 90 to 50% 0.1 M NaOH and from 10 to 50% 0.1 M NaOH/1 M NaOAc at a flow rate of 0.2 mL min^−1^, and then further extended to 100% 0.1 M NaOH/1 M NaOAc over 33 min at the same flow rate and kept for 8 min. The gradient was then reverted to 90% 0.1 M NaOH and 10% 0.1 M NaOH/1 M NaOAc in 0.2 min at a flow rate of 0.25 mL min^−1^ and kept for 7 min before starting the next run. Mono- and oligo-galacturonides were identified by comparison to retention times of external standards (Megazyme) and quantified by comparing integrated peak areas of samples to those of calibration standards each at 125, 250 and 500 µM. Oxidised short OGs were used to assign retention times to oxidised long OGs.

### Analysis of native and oxidised OGs by NMR spectroscopy

Fifteen mg of commercial oligogalacturonides DP10-15 (Elicityl) were treated with 380 nM *Pi*AA7A BBE in 5 mL reactions containing 4 mM sodium phosphate buffer, pH 7, for 24 h at 20 °C. The reaction was treated with 1 mL ice-cold ethanol to precipitate the enzyme and centrifuged at 14800 × *g* for 10 min at 4 °C. The supernatant was then collected, and the protein pellet resuspended in a further 1 mL ice-cold EtOH and centrifuged again at 14,800 × *g* for 10 min at 4 °C. The supernatants were combined and the EtOH content of the sample was diluted to below 10% before lyophilisation to afford the oxidised oligosaccharide as a colourless foam, which was characterised by NMR spectroscopy: ^1^H-NMR (700 MHz, D_2_O): 5.06 (br d, 1H, H1’), 4.96 (br d, ~9H, H1”), 4.94 (d, 1H, H1”‘, *J*_1”‘,2”‘_ = 3.8 Hz), 4.67 (m, ~10H, H5”, H5”‘), 4.31 (m, ~9H, H4”), 4.28 (m, 1H, H5’), 4.21 (m, 1H, H5 or H2), 4.19 (m, 1H, H4’) 4.15 (m, 1H, H4”‘), 4.06 (m, 1H, H5 or H2), 3.93 (m, 1H, H3’), 3.90 (dd, ~9H, *J*_2”,3”_ = ~10 Hz, *J*_3”,4”_ = ~3 Hz, H3”), 3.80 (dd, 1H, *J*_2”‘,3”‘_ = 10.4 Hz, *J*_3”‘,4”‘_ = 3.2 Hz, H3”‘), 3.72 (dd, 1H, *J*_2’,3’_ = 10.7 Hz, *J*_1’,2’_ = 4 Hz, H2’), 3.69-3.64 (m, ~11H, H2”, H3, H4), 3.60 (dd, 1H, *J*_2”‘,3”‘_ = 10.3 Hz, *J*_1”‘,2”‘_ = 3.8 Hz, H2”‘); ^13^C-NMR (175 MHz, D_2_O): 179.8, 179.4 (C1, C6), 177.9 (C6”‘), 175.3 (C6”), 174.8 (C6’), 98.8 (C1”, C1”‘), 96.9 (C1’), 77.6 (C4”), 76.3 (C4’), 72.0, 71.9 (C3, C4), 71.4 (C5’), 71.1 (C5”, C5”‘, C3’, C2 or C5), 70.7 (C4”‘), 69.9 (C2 or C5), 69.3 (C3”‘), 68.6 (C3”) 68.1 (C2”‘), 67.9 (C2”), 67.6 (C2’).

### Oxidative burst experiments using long OGs (DP10-15, Elicityl)

Oxidised long OGs were prepared by mixing 150 nM *Pi*AA7A with 2 mg mL^−1^ long OGs (DP10-15, Elicityl) in 50 mM Tris-HCl pH 7 in a total reaction volume of 25 mL, incubated on a gyro-rocker at 20 °C for 16 hours before being filtered through 30 kDa centrifugal columns (Amicon Ultra-15, Millipore). For non-enzyme controls, long OGs were incubated in buffer only and treated as above. Four mm leaf discs were collected from pre-flowering 4 to 5 weeks-old *A. thaliana* Col-0 and *Solanum lycopersicum* cv. MicroTom, germinated in soil and grown in a controlled chamber under a photoperiod of 16 hours light (100 µmol photons m^−2^ s^−1^)/8 h darkness. For *A. thaliana*, the number of replicates corresponded to 6 leaves (*n* = 6) for mock, 12 leaves (*n* = 12) for OG, 8 leaves (*n* = 8) for OGox, and 5 leaves (*n* = 5) for OG + OGox. Only one disc was taken from a leaf, and only one leaf was taken from an individual plant. For cv. MicroTom, 6 leaves (*n* = 6) were used for OG, OGox and mock, respectively, and 5 leaves (*n* = 5) for OG + OGox. After being floated on water overnight, leaf discs were transferred into a 96-well plate, and water was changed twice. Water was then replaced with 100 µl solution of 34 µg mL^−1^ (w/v) luminol (Sigma), 20 µg mL^−1^ horseradish peroxidase (Sigma) and Tween20 at 0.0001% (w/v) in 10 mM potassium phosphate buffer pH 7.5, and containing either 0.2 mg mL^−1^ OG or OGox (individually), or 0.2 mg mL^−1^ OG and 0.2 mg mL^−1^ OGox (OG + OGox), or water for mock experiment. The plate was transferred to the Tecan infinity 200 plate reader set in luminescence mode for relative light emission quantification^45^.

### Reporting summary

Further information on research design is available in the Nature Portfolio Reporting Summary linked to this article.

## Supplementary information

Supplementary Information

Description of Additional Supplementary Files

Supplementary Dataset 1

Supplementary Dataset 2

Supplementary Dataset 3

Supplementary Dataset 4

Reporting Summary

Transparent Peer Review file

## Source data

Source data

## Data Availability

Data generated in this study are provided in the main manuscript, figures, supplemental figures, and in source data files. Source data are provided in this paper.
