# Supplementary material for: Oomycetes manipulate plant innate immunity through galacturonide oxidases
Source: Nat Commun. 2025 Oct 20;16:9093. doi: 10.1038/s41467-025-64189-1 (PMC12537922; doi:10.1038/s41467-025-64189-1)
Supplement: Supplementary file 1 — Supplementary Information [file 41467_2025_64189_MOESM1_ESM.pdf]

Supplementary Information for:

Oomycetes manipulate plant innate immunity through galacturonide oxidases

Lydia R. J. Welsh[1#], Anna O. Avrova[1#], Katrin Besser[2], Talia Kirkbride[2], Carla Botelho Machado[2], Natasha E. Hatton[3], Leonardo D. Gomez[2], Martin A. Fascione[3], Jared Cartwright[4], Petra C. Boevink[1], Katherine Denby[2], David Cannella[5], Simon J. McQueen-Mason[2†], Stephen C. Whisson[1] and Federico Sabbadin[2]*

Affiliations:

[1]Cell and Molecular Sciences, James Hutton Institute, Invergowrie, Dundee, UK.

[2]Centre for Novel Agricultural Products, Department of Biology, University of York, York, UK.

[3]Department of Chemistry, University of York, York, UK.

[4]Bioscience Technology Facility, Department of Biology, University of York, York, UK.

[5] PhotoBiocatalysis Unit, Crop Production and Biostimulation Lab, Universitè libre de Bruxelles, Brussels, Belgium.

[#]These authors contributed equally.

*Corresponding author. Email: federico.sabbadin@york.ac.uk

[†] Author deceased before the submission of this manuscript

Supplementary Figures

Supplementary Fig. 1: Stereo view of the aromatic cluster involved in stabilising the catalytic base in selected AA7s.
Supplementary Fig. 2: Lack of conservation of the aromatic residue usually involved in stacking onto the neutral saccharide unit penultimate to the reducing end.
Supplementary Fig. 3: Electrostatic surface potential of *Phytophthora* AA7s.
Supplementary Fig. 4: Purification, thermal shift assays and UV-vis analysis of recombinant *P. infestans* AA7s produced in *P. pastoris*.
Supplementary Fig. 5: Time course activity assays of *P. infestans* AA7s incubated with commercial OGs.
Supplementary Fig. 6: MALDI-TOF MS spectra (positive mode) of products released by *Pi*AA7B-C upon incubation with long OGs (DP10-15, Elicityl).
Supplementary Fig. 7: NMR analysis of long OGs (DP10-15, Elicityl) following oxidation by *Pi*AA7A.
Supplementary Fig. 8: Determination of pH optima for *P. infestans* AA7s using commercial OGs.
Supplementary Fig. 9: Further representative images of *Pi*AA7A localisation during infection of *N. benthamiana* leaves with *P. infestans*.
Supplementary Fig. 10: Representative images of *Pi*AA7A localisation during infection of potato leaves with *P. infestans*.
Supplementary Fig. 11: Western blot of *Pi*AA7A-mCherry fusion expressed in *P. infestans*.
Supplementary Fig. 12: Multiple sequence alignment of *Pi*AA7 sequences and selected region for gene silencing.
Supplementary Fig. 13: Growth of *P. infestans* silenced lines in artificial medium.
Supplementary Fig. 14: Analysis of correlation between expression of *PiAA7A-E* genes and lesion size across all silenced *P. infestans* lines infecting potato leaves (5 dpi).
Supplementary Fig. 15: Phylogeny and surface charges of AA7 proteins found in *Arabidopsis thaliana*, *Solanum lycopersicum* and *Solanum tuberosum*.
Supplementary Fig. 16: Wider phylogeny of AA7 proteins across oomycetes, *Arabidopsis* and selected fungal species.

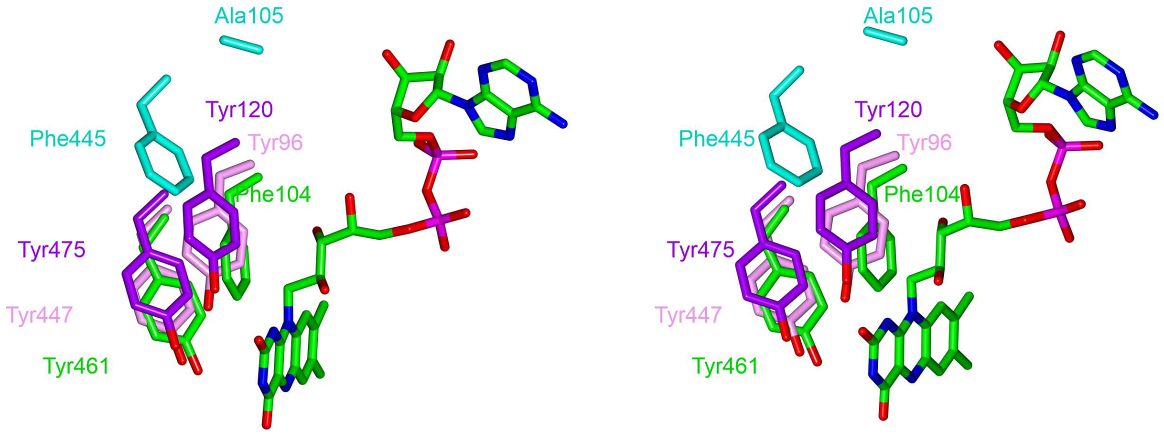

Supplementary Fig. 1 | Stereo view of the aromatic cluster involved in stabilising the catalytic base in selected AA7s. *Pi*AA7A (green, representing Clade I), UniProt sequence G4ZRJ9 from *P. sojae* (purple, representing Clade II), UniProt sequence A0A1V9ZHL9 from *Achlya hypogyna* (cyan, representing Clades III-IV) and chitooligosaccharide oxidase (ChitO, PDB file 6Y0R) from *Fusarium graminearum* (pink), following superimposition of all models relative to ChitO. In Clades III-IV, the aromatic cluster is not conserved, and residue 105 is either Ala (as shown in the figure) or Pro, and residue 445 is Phe (as shown in the figure) or His or Ile. All oomycete AA7 3D models were created using AlphaFold3. See Methods for details on the selection of Clade I-IV representatives and the generation of 3D structural models.

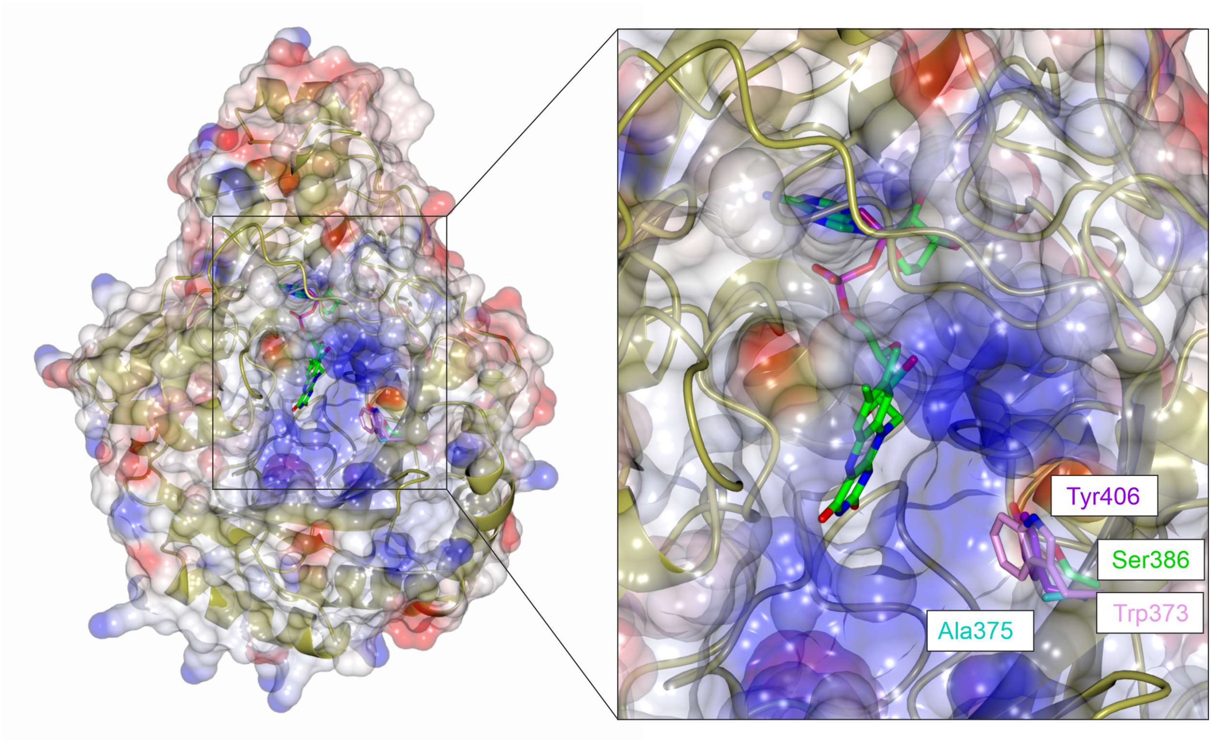

Supplementary Fig. 2 | Lack of conservation of the aromatic residue usually involved in stacking onto the neutral saccharide unit penultimate to the reducing end. *Pi*AA7A (green, representing Clade I), UniProt sequence G4ZRJ9 from *P. sojae* (purple, representing Clade II), UniProt sequence A0A1V9ZHL9 from *Achlya hypogyna* (cyan, representing Clades III-IV) and chitooligosaccharide oxidase (ChitO, PDB file 6Y0R) from *Fusarium graminearum* (pink), following superimposition of all models relative to *Pi*AA7A. An aromatic residue is only found in ChitO (Trp373) and Clade II (exemplified by Tyr406 in G4ZRJ9) but not in Clade I (Ser386 in *Pi*AA7A) and Clade III-IV (A375 in A0A1V9ZHL9). Models for Clade I, II and III-IV were created using AlphaFold3. See Methods for details on the selection of Clade I-IV representatives and the generation of 3D structural models.

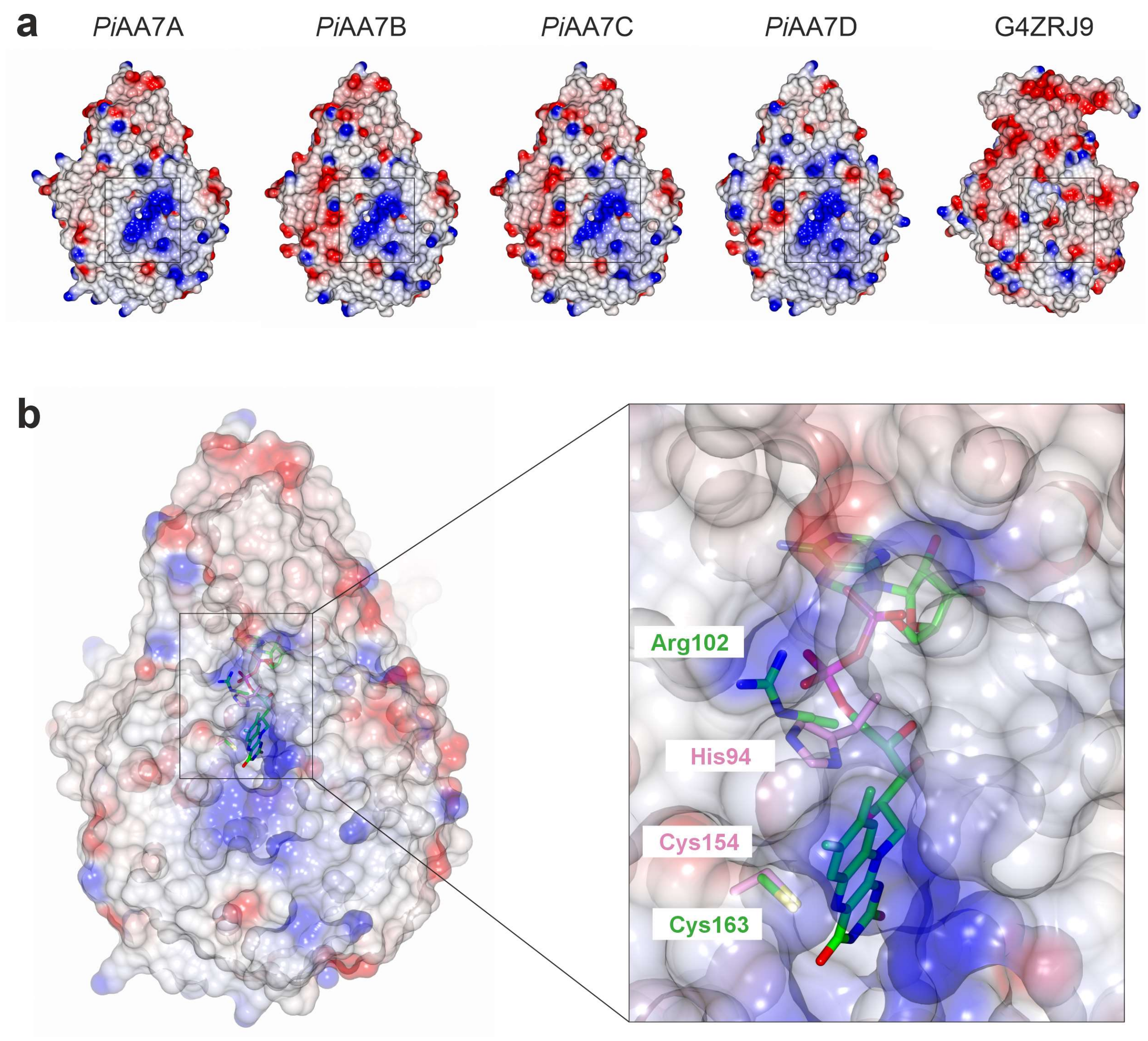

Supplementary Fig. 3 | Electrostatic surface potential of *Phytophthora* AA7s. a) Highly conserved patch of positively charged residues (arginines and lysines) surrounds the opening leading to the solvent-exposed FAD cofactor, suggesting specific interactions with negative charged substrates. This feature is found in Clade I AA7s (exemplified by *Pi*AA7A-D from *P. infestans*), but not in Clade II (exemplified by G4ZRJ9 from *P. sojae*). **b)** The highly conserved histidine involved in 8α-N1-histidylation of FAD in canonical AA7s, as well as in Clades II and III-IV, is exemplified here by His94 from chitooligosaccharide oxidase (ChitO from *Fusarium graminearum*, PDB file 6Y0R, pink). This residue is replaced by an arginine in all Clade I oomycete AA7s (Arg102 in *Pi*AA7A). See Methods for details on the selection of Clade I-IV representatives and the generation of 3D structural models.

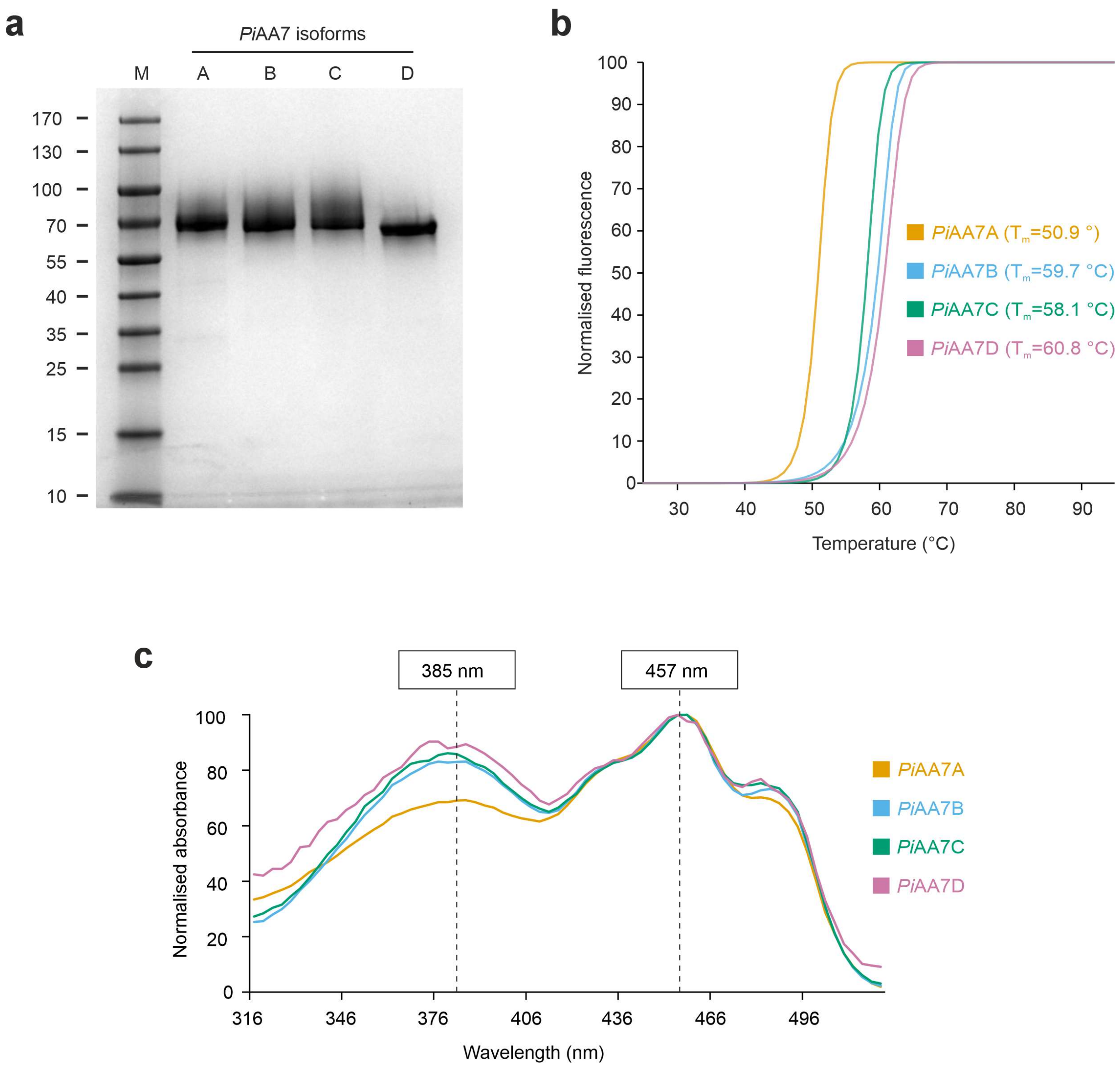

Supplementary Fig. 4 | Purification, thermal shift assays and UV-vis analysis of recombinant *P. infestans* AA7s produced in *P. pastoris*. a) SDS-PAGE analysis of purified 6-his-tagged proteins following Ni-NTA and size exclusion chromatography. M: protein marker. A-D correspond to the four AA7 isoforms from this study. Numbers on the left indicate the molecular weight in kDa. The correct identity of the bands was confirmed through protein ID by mass spectrometry (see Methods). **b)** Melting curves of the purified AA7s, highlighted with different colours. T_m: melting temperature. **c)** UV-VIS spectra of purified AA7s, showing FAD peaks at 385 and 457 nm.

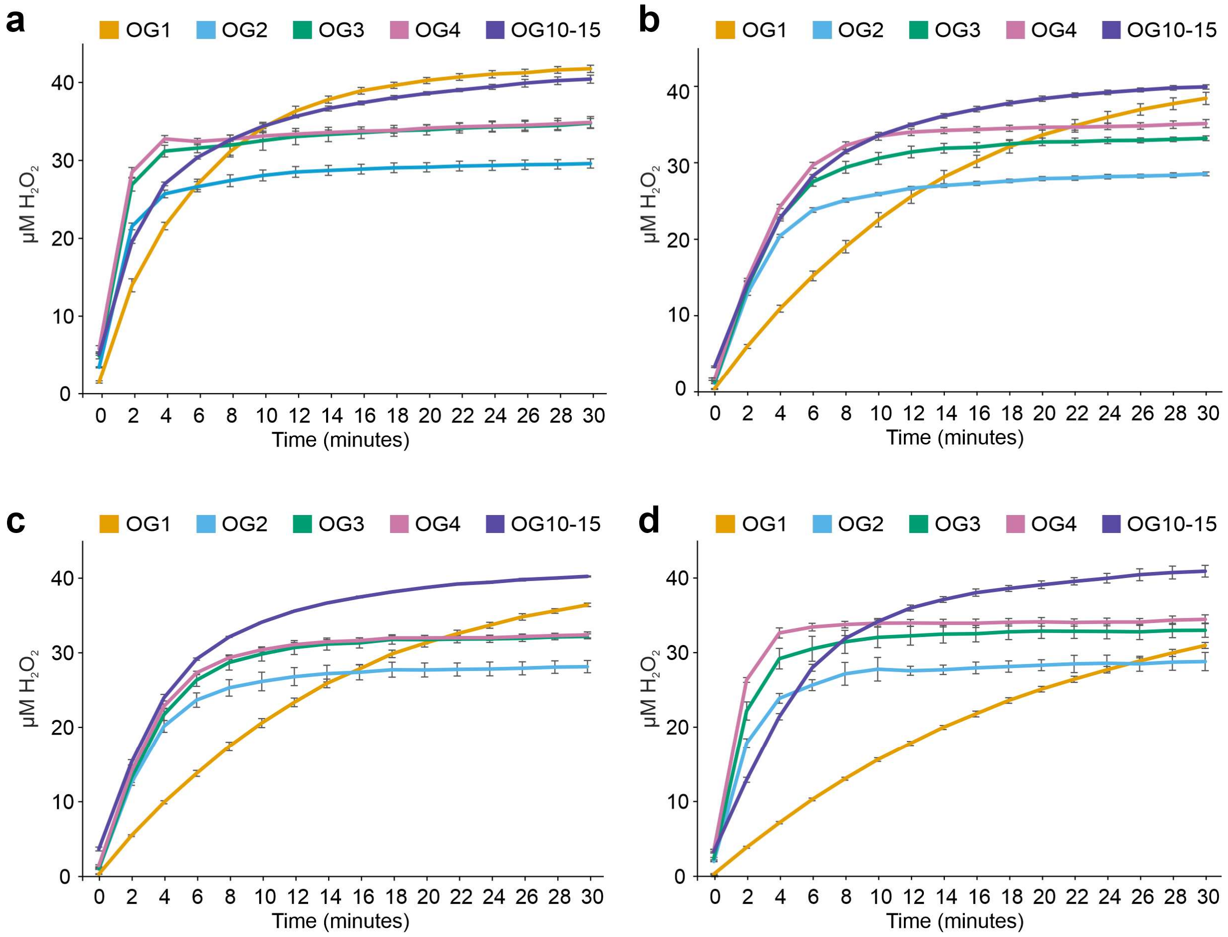

Supplementary Fig. 5 | Time course activity assays of *P. infestans* AA7s incubated with commercial OGs. Time course analysis of OG oxidising activity of *Pi*AA7A **(a)**, *Pi*AA7B **(b)**, *Pi*AA7C **(c)** and *Pi*AA7D **(d)** using a peroxidase-coupled colourimetric assay with commercial OGs of DP 1 to 4 (Megazyme), and DP10-15 (Elicityl). Reactions were run for 30 min in 25 mM SHAM buffer at pH 6, measuring absorbance at 514 nm every 2 min on a BMG SpectrostarNano microplate reader. For short OGs (DP1 to 4, Megazyme) 0.05 mM substrate was incubated with 10 nM enzyme, while for long OGs (DP10-15, Elicityl), reactions were carried out using 0.2 mg mL^{-1} substrate and 5 nM enzyme. All experiments were run in triplicate, average values shown. Error bars: standard deviations of three replicates (n = 3).

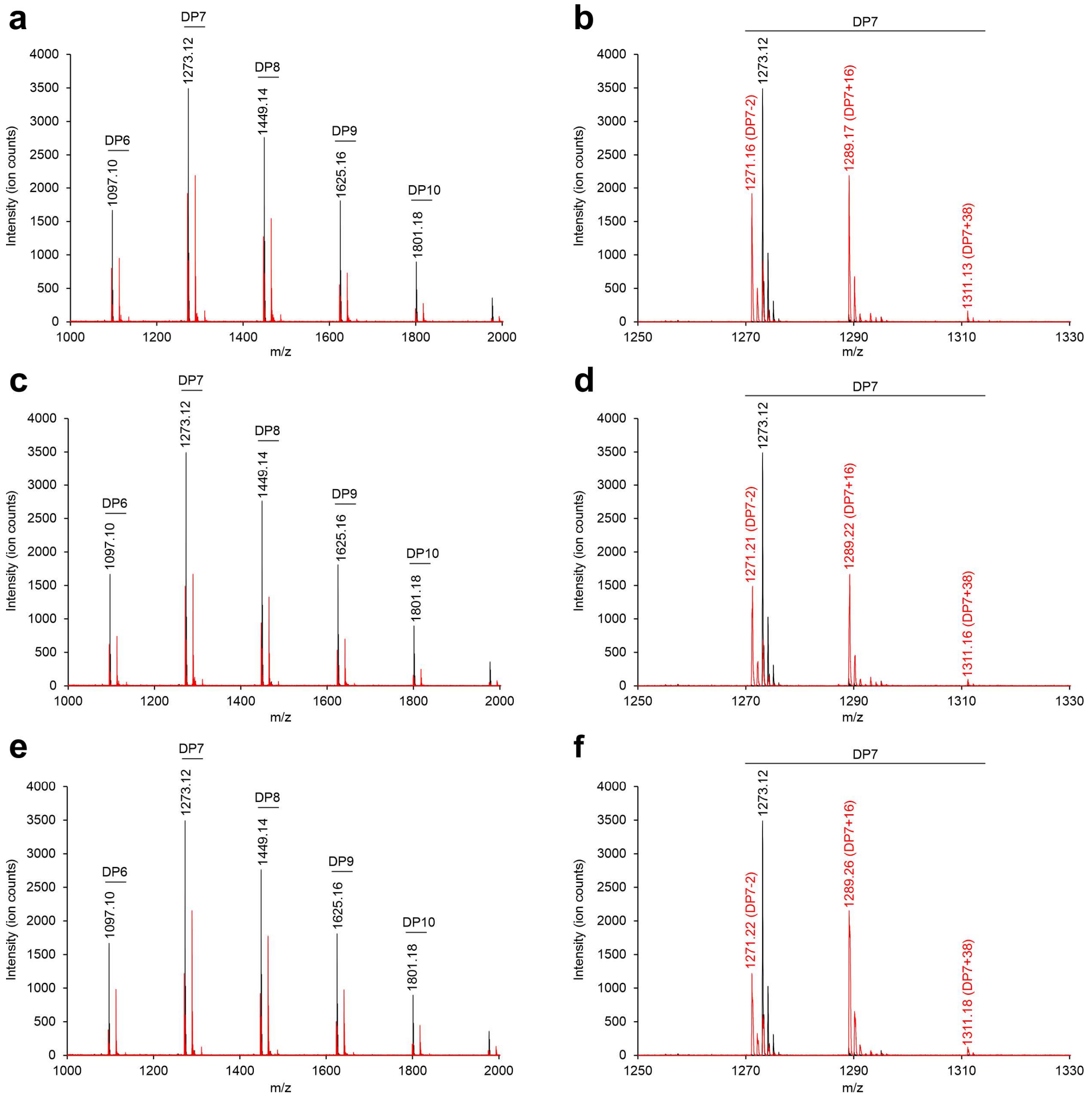

Supplementary Fig. 6 | MALDI-TOF MS spectra (positive mode) of products released by *Pi*AA7B-C upon incubation with long OGs (DP10-15, Elicityl). 600 nM *Pi*AA7B (panels **a, b**), *Pi*AA7C (panels **c, d**) and *Pi*AA7D (panels **e, f**) was incubated with 10 mg mL^{-1} long OGs (commercially supplied as average DP10-15, Elicityl, also containing OGs with DP 6-9) in 20 mM ammonium acetate buffer pH 6 for 12 hours 20 °C. The panels show superimposed substrate (black line) and products (red line) spectra. Panels **b**, **d** and **f** show expanded spectra for DP7 upon incubation with *Pi*AA7B, C and D, respectively. Native and oxidised species are labelled in black and red, respectively. The main peaks correspond to mono- or di-sodiated adducts. C1-keto form (-2 species), and C1 aldonic acids, imparting +16 or +38 *m/z* relative to the mono-sodiated unoxidised form, are marked in red. E.g. panel b: *m/z* 1271.16: -2 species, oxidised (ketone, mono-sodiated). *m/z* 1273.12: native species (mono-sodiated). *m/z* 1289.17: +16 species, oxidised (aldonic acid, mono-sodiated). *m/z* 1311.13: +38 species, oxidised (aldonic acid, di-sodiated).

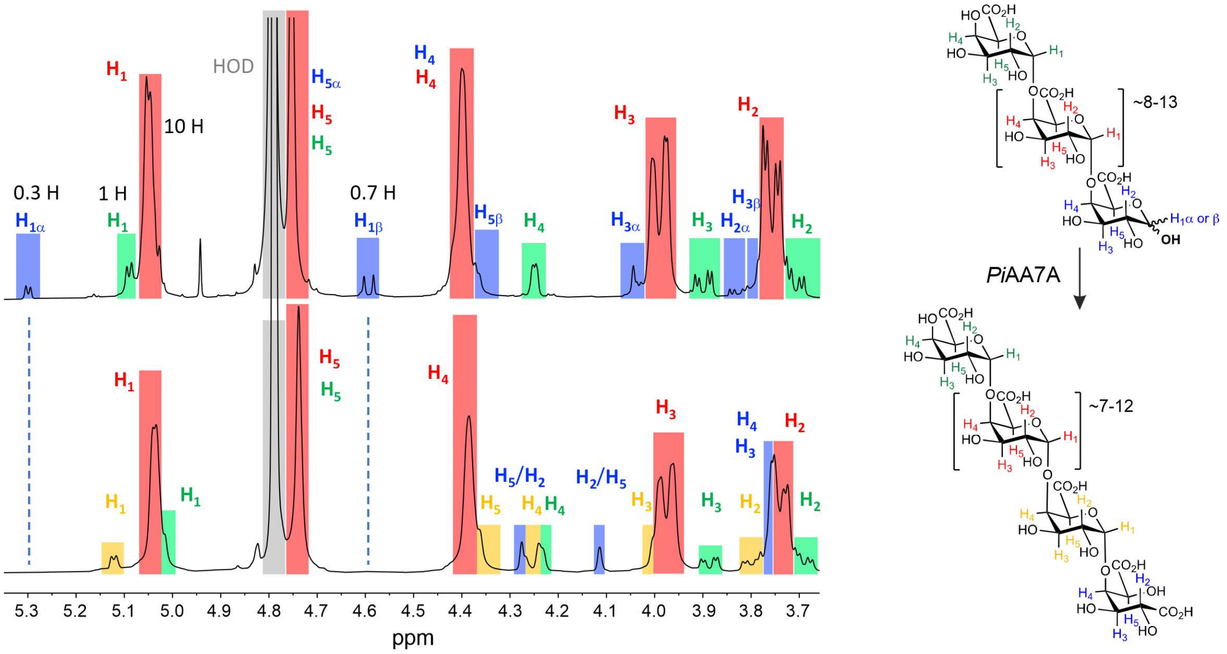

Supplementary Fig. 7 | NMR analysis of long OGs (DP10-15, Elicityl) following oxidation by *Pi*AA7A. 500 MHz D_2O ^{1}H-NMR of oligogalacturonic acid (commercially supplied as average DP10-15) prior (top) and following (bottom) oxidation of 3 mg mL^{-1} substrates with 380 nM *Pi*AA7A in 4 mM sodium phosphate buffer pH 7, for 24 hours at 20 °C. Blue dashed lines indicate loss of H1α and β of reducing sugar (blue) indicative of C1 oxidation.

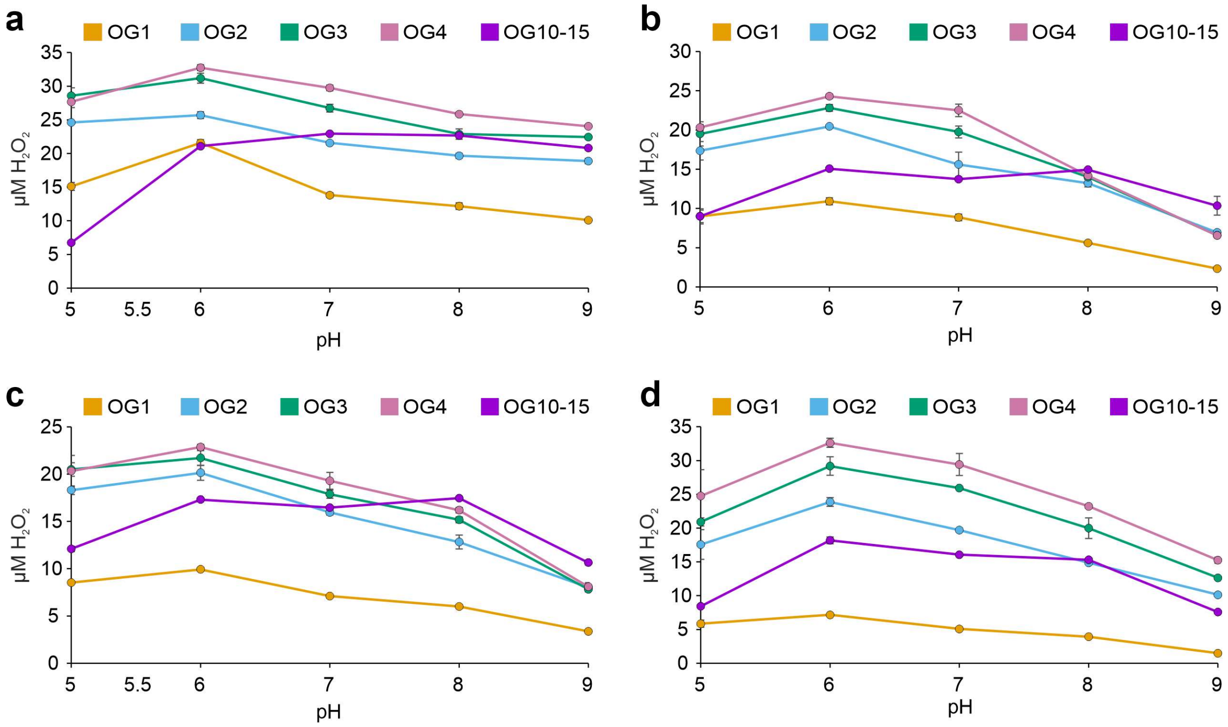

Supplementary Fig. 8 | Determination of pH optima for *P. infestans* AA7s using commercial OGs. Activity of *Pi*AA7A **(a)**, *Pi*AA7B **(b)**, *Pi*AA7C **(c)** and *Pi*AA7D **(d)** was determined through a peroxidase-coupled colourimetric assay, using commercial short OGs of DP1 to 4 (Megazyme), and long OGs with DP10-15 (Elicityl). Reactions were run for 4 min in 25 mM of either SHAM buffer at pH 5-8, or Tris-HCl buffer at pH 9, plus 10 nM enzyme and 0.05 mM OG1/2/3/4 (Megazyme), measuring absorbance at 514 nm on a BMG SpectrostarNano microplate reader. For long OGs, reactions were carried out using 0.2 mg mL^{-1} substrate and 5 nM enzyme. All experiments were run in triplicate, average values shown. Error bars: standard deviations of three replicates (n = 3).

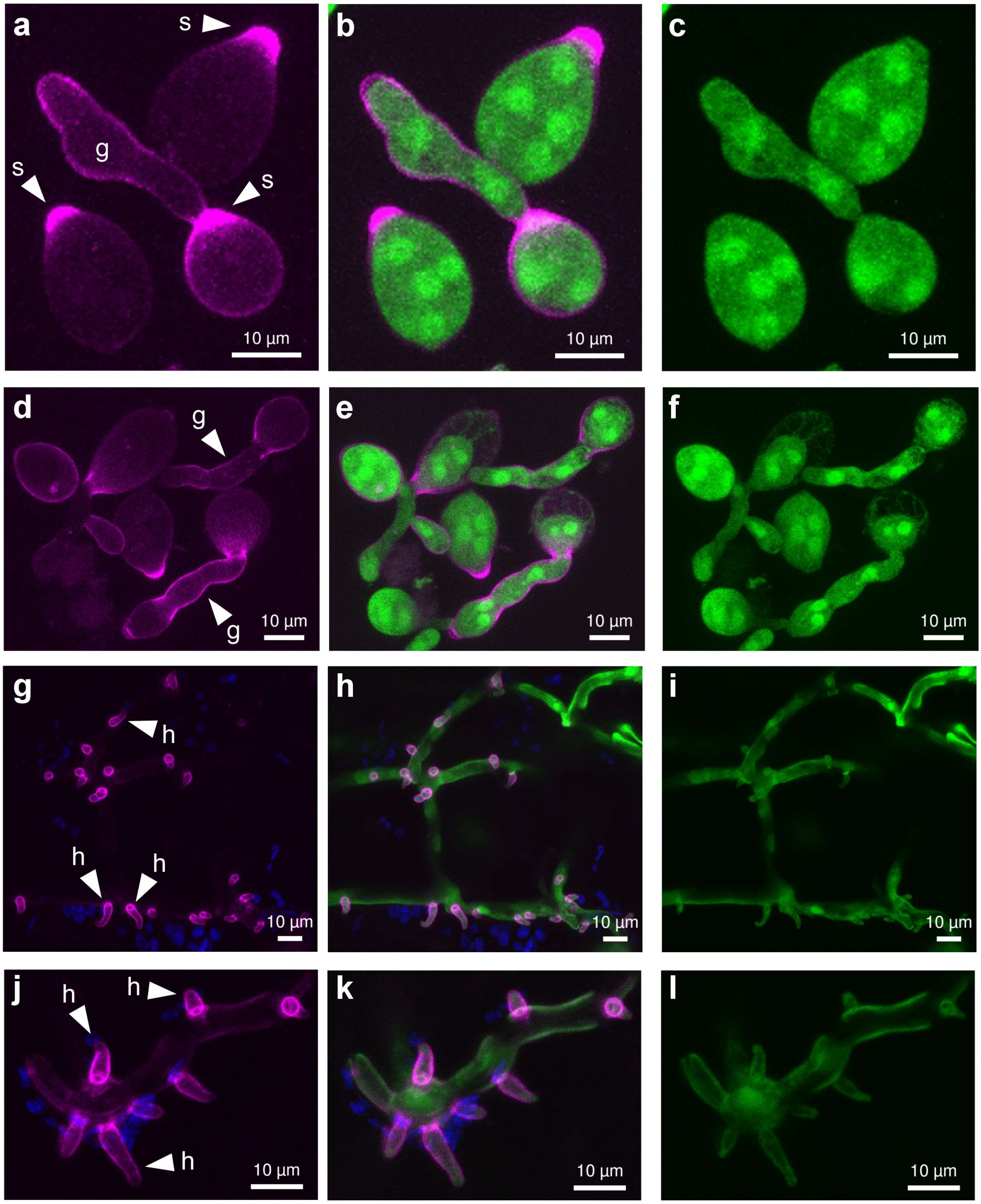

Supplementary Fig. 9 | Further representative images of *Pi*AA7A localisation during infection of *N. benthamiana* leaves with *P. infestans*. Confocal projection images of *P. infestans* isolate 2006-3928A expressing a *Pi*AA7A-mScarlet fusion protein (pink) under the control of the native *PiAA7A* promotor and cytoplasmic mCitrine (green). *Pi*AA7A-mScarlet localises to tips of sporangia (s) and periphery of sporangia and germ tubes (g) on the surface of an *N. benthamiana* leaf **(a, b, d, e)**. In infectious hyphae the fusion locates to haustoria (h), especially at the base and periphery of the hyphae **(g, h, j, k)**. Panels **g, h, j,**

k also show chlorophyll autofluorescence from the plant cells in blue. Images **a-f** and **g-l** were collected at three and four days post inoculation (dpi), respectively. Images shown are: mScarlet in pink **(a, d, g, j)**, merged images with mScarlet in pink and mCitrine in green **(b, e, h, k)**, cytoplasmic mCitrine in green **(c, f, i, l)**. To aid interpretation, selected sporangial tips (s), germ tubes (g), and haustoria (h) are labelled in the images. Scale bars indicate 10 μm. Each image is representative of at least 10 images of independent infection points.

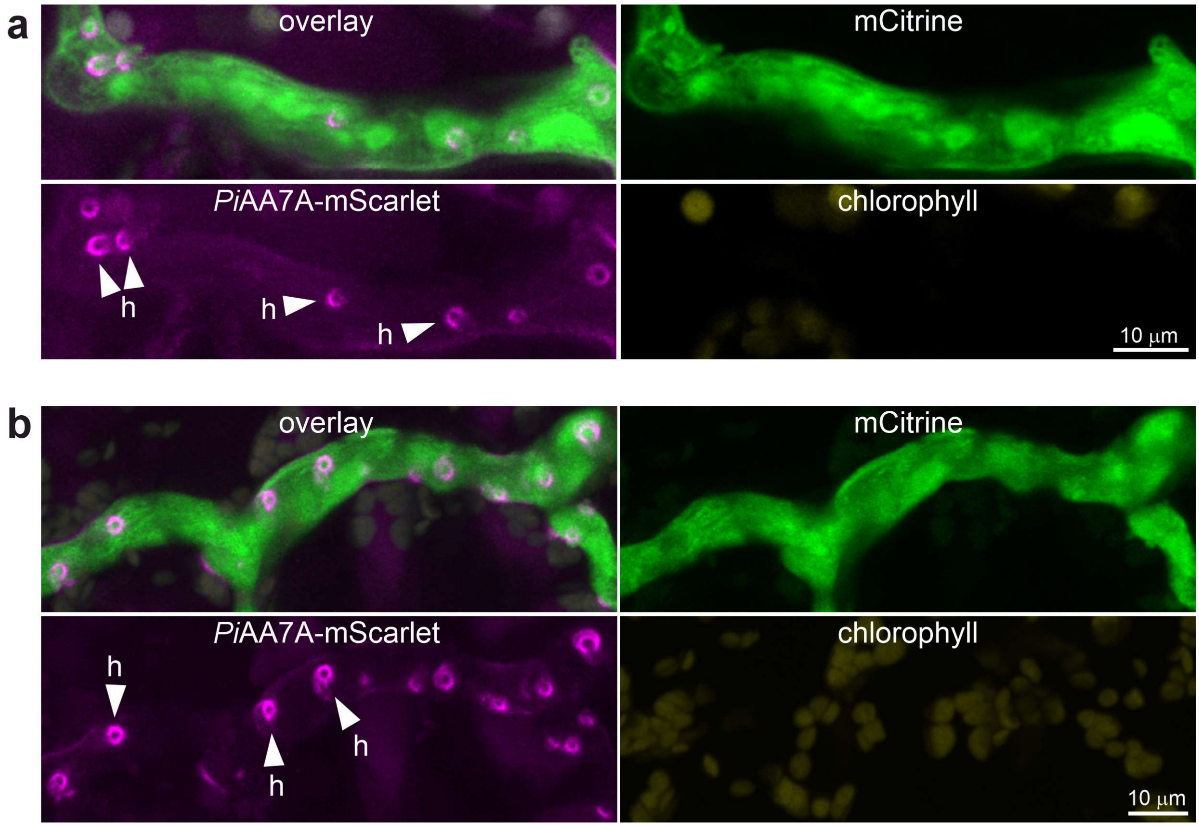

Supplementary Fig. 10 | Representative images of *Pi*AA7A localisation during infection of potato leaves with *P. infestans*. **a** and **b** are independent confocal projection images of *P. infestans* isolate 2006-3928A expressing a *Pi*AA7A-mScarlet fusion protein (pink) under the control of the native *PiAA7A* promotor and cytoplasmic mCitrine (green). In infectious hyphae the fusion locates to haustoria (h), especially at the base, and periphery of hyphae. Chlorophyll autofluorescence from the plant cells is shown in yellow. Images were collected from the leading edges of expanding disease lesions at four days post inoculation (dpi). Example haustoria are indicated in the images with an arrowhead and letter h. Scale bars indicate 10 μm. Each image is representative of at least 10 images of independent infection points.

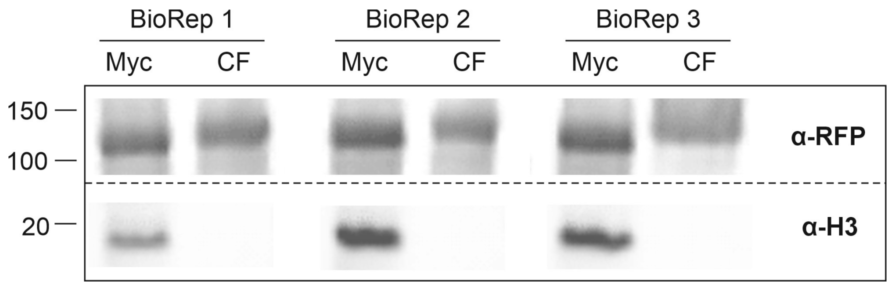

Supplementary Fig. 11 | Western blot of *Pi*AA7A-mCherry fusion expressed in *P. infestans*. The mCherry fusion protein was detected in both mycelia (Myc) and culture filtrate (CF) using a mouse α-RFP antibody (6G6 ProteinTech) and goat anti-mouse antibody conjugated to IRdye 800CW (LICORbio). Histone H3 was detected only in mycelia using a rabbit α-histone H3 antibody (Abcam) and IR dye 680RD-conjugated goat anti-rabbit antibody (LICORbio), indicating no detectable cell lysis during the experiment. The results for three biological replicates (BioRep 1, 2 and 3) are shown. Numbers on the left indicate the molecular weight in kDa.

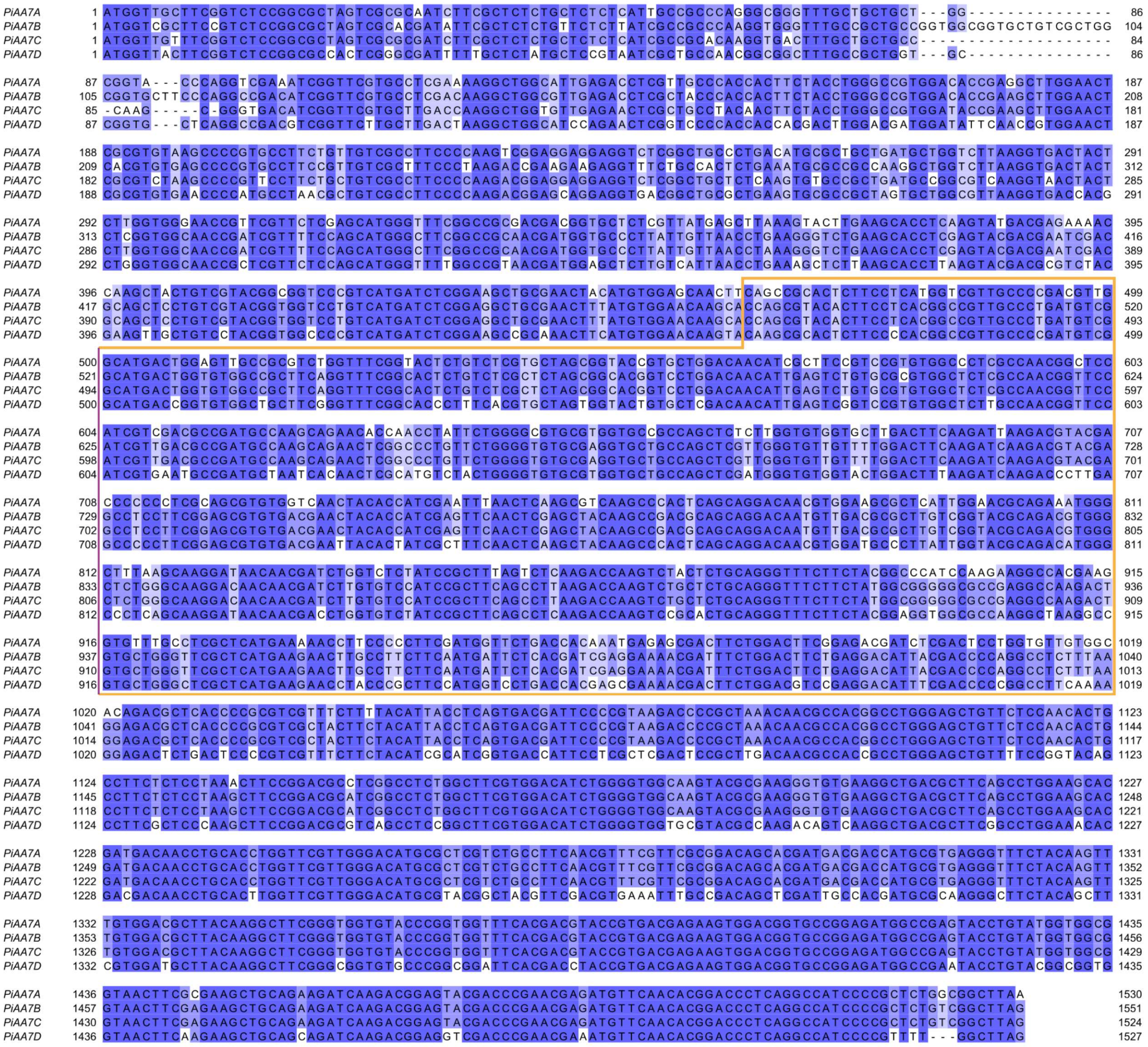

Supplementary Fig. 12 | Multiple sequence alignment of *PiAA7* sequences and selected region for gene silencing. A 508 bp fragment of *PiAA7A* is highlighted in an orange box, representing a conserved part of the DNA sequence that was PCR amplified from genomic DNA of *P. infestans* isolate 2006_3928A.

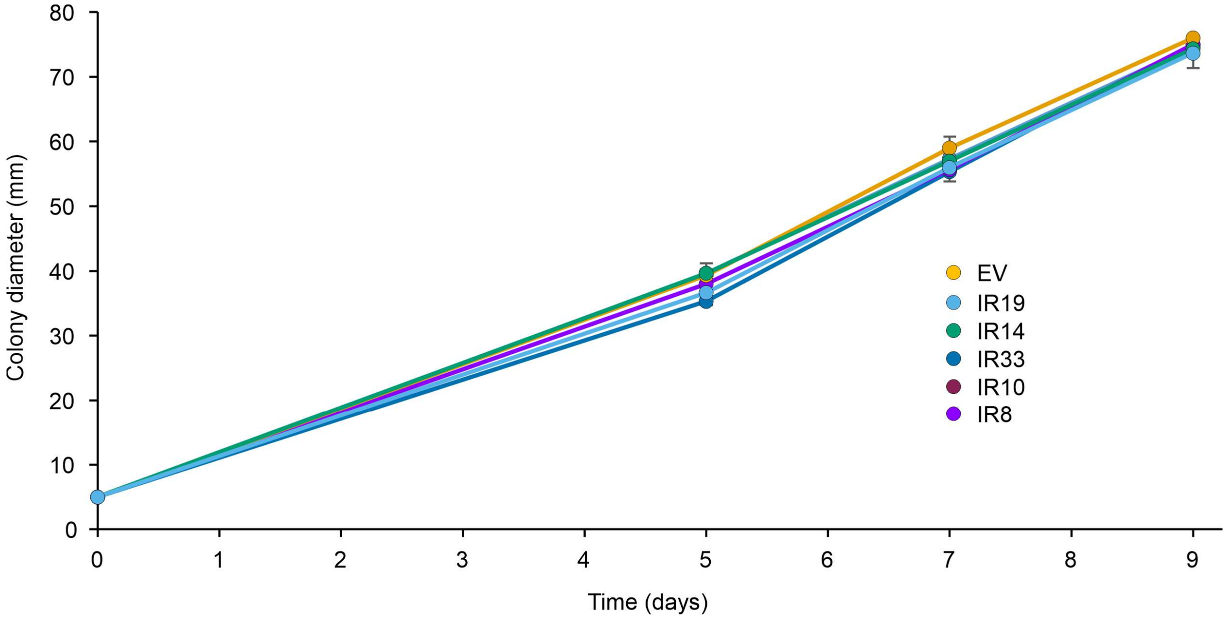

Supplementary Fig. 13 | Growth of *P. infestans* silenced lines in artificial medium. Recovered transformed lines (IR8, IR10, IR14, IR19 and IR33) were grown on rye-sucrose agar medium by measuring colony diameter over time, compared to the empty vector (EV). All experiments were done in triplicate, average values shown. Error bars: standard deviations of three replicates ($n = 3$). Note that the IR10 trace is not visible as it is obscured by IR14, which has a near identical profile.

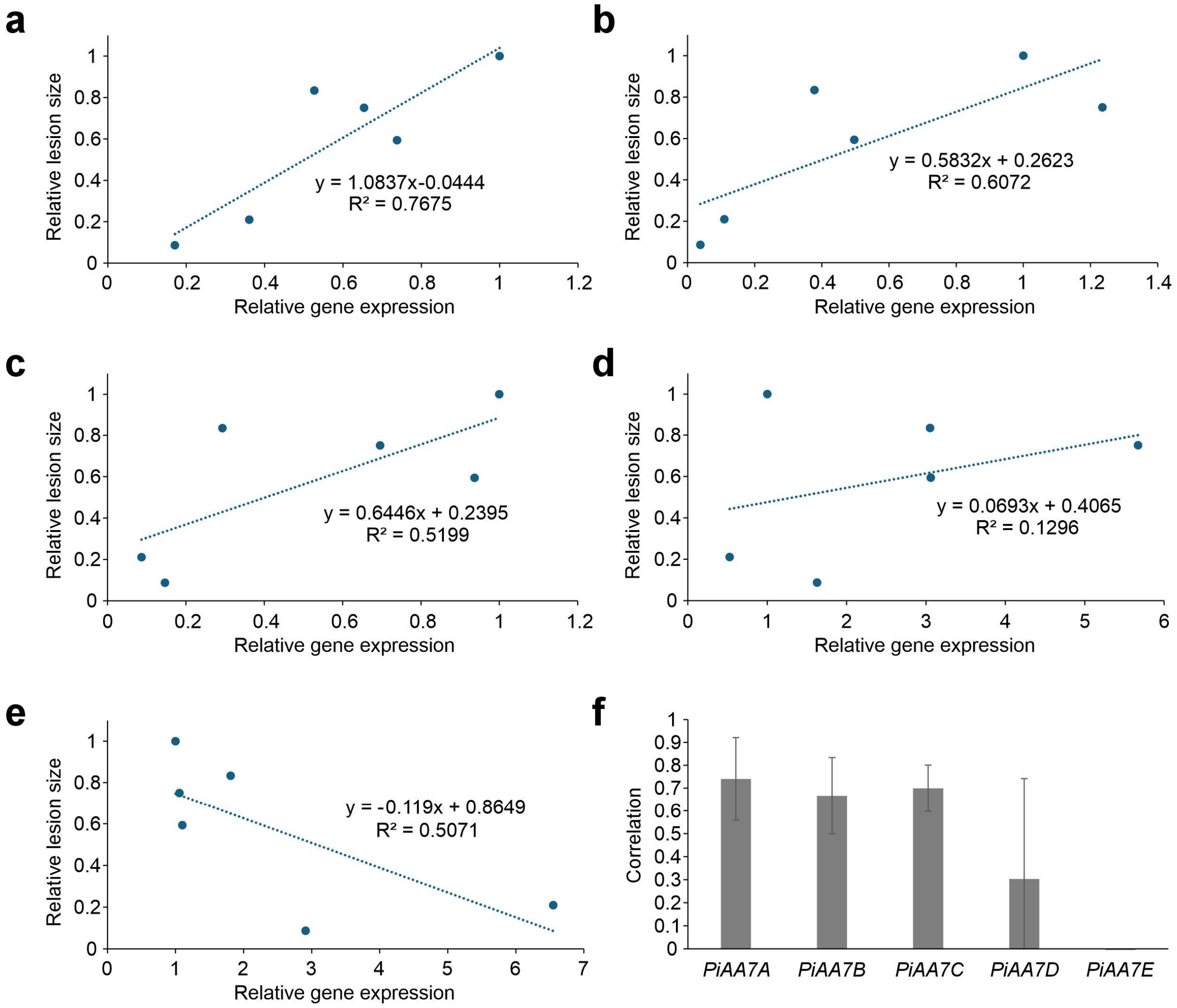

Supplementary Fig. 14 | Analysis of correlation between expression of *PiAA7A-E* genes and lesion size across all silenced *P. infestans* lines infecting potato leaves (5 dpi). a-e) Each panel shows the analysis for a single gene. **a**: *PiAA7A*. **b**: *PiAA7B*. **c**: *PiAA7C*. **d**: *PiAA7D*. **e**: *PiAA7E*. Each dot represents the average value of three biological replicates for each silenced line (IR8, IR10, IR14, IR19 and IR33) relative to empty vector (EV) control. **f**) Correlation between expression of *PiAA7A-E* and lesion size, both relative to EV control, across all silenced *P. infestans* lines. Values indicate means (error bars: standard deviations of three replicates, $n = 3$).

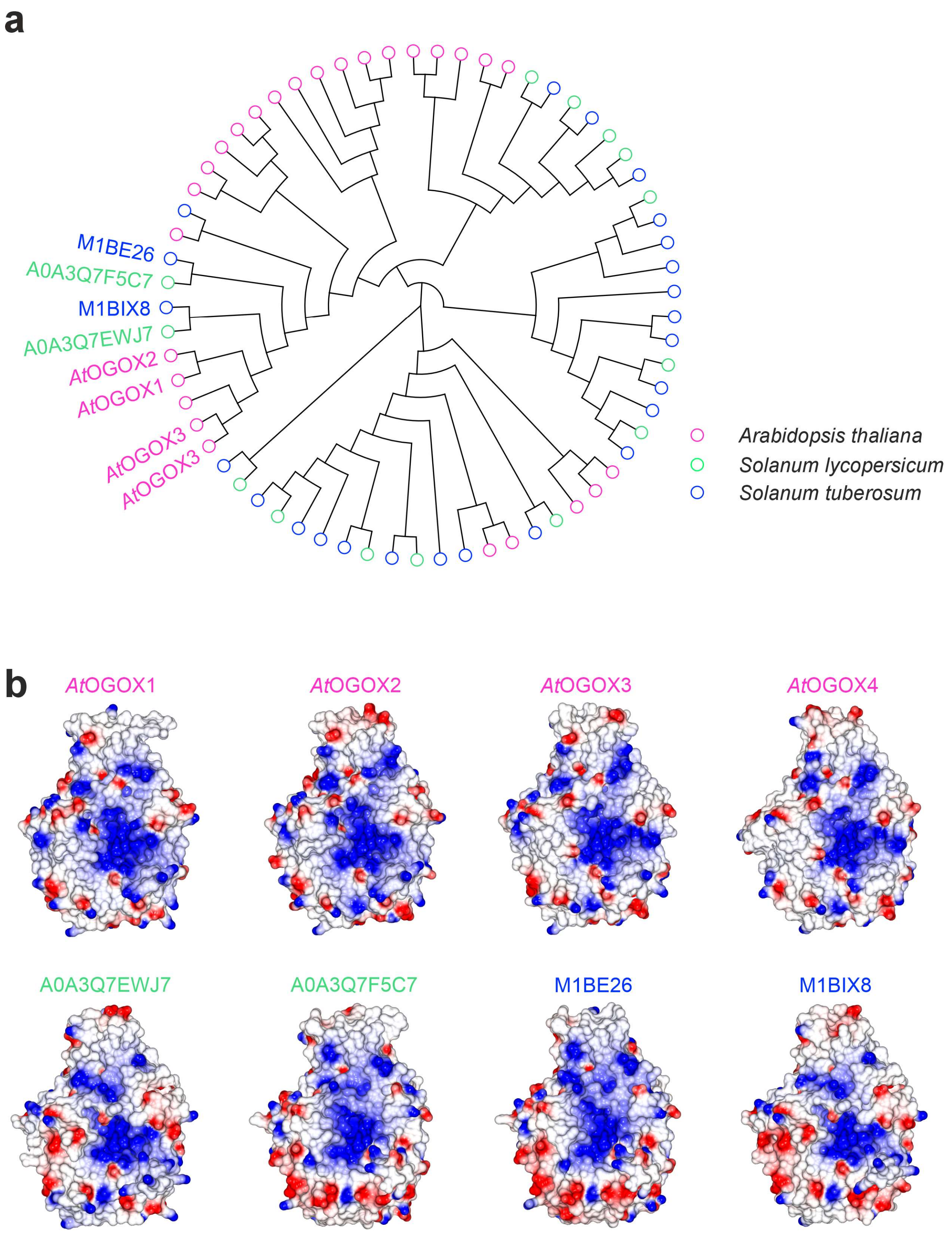

Supplementary Fig. 15 | Phylogeny and surface charges of AA7 proteins found in *Arabidopsis thaliana*, *Solanum lycopersicum* and *Solanum tuberosum*. a) Neighbour-joining tree showing *A. thaliana*, *S. lycopersicum* and *S. tuberosum* AA7s in pink, green and blue, respectively. The four characterised OGOX isoforms from *Arabidopsis* are labelled, alongside closely related orthologues from *S. lycopersicum*

and *S. tuberosum* (see Methods for more details). **b)** Electrostatic surface potential of AlphaFold3 models of proteins highlighted in panel a, with positively charged residues (in blue) marking the boundaries of the entrance of the active site.

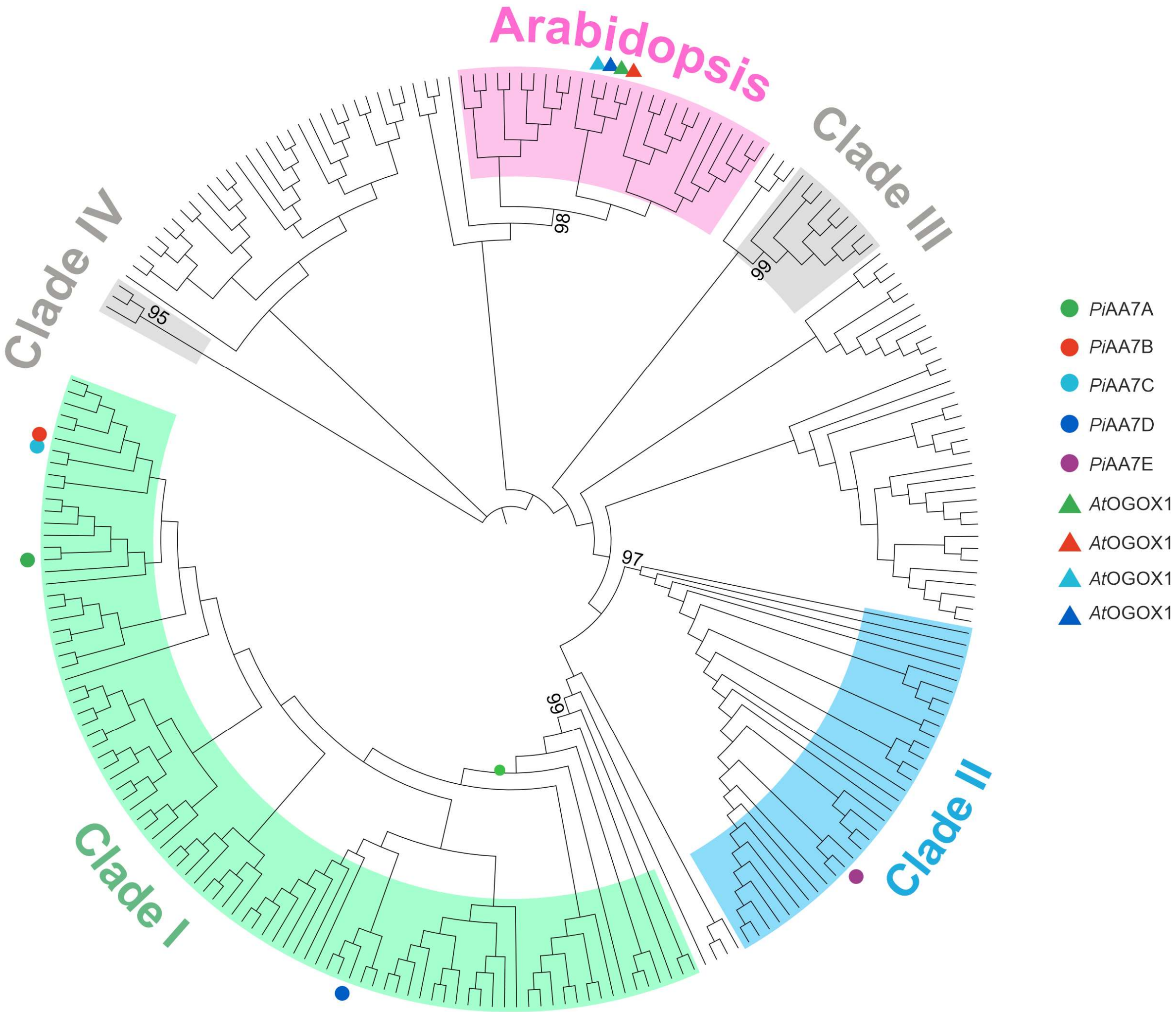

Supplementary Fig. 16 | Wider phylogeny of AA7 proteins across oomycetes, *Arabidopsis* and selected fungal species. Neighbour-joining tree showing oomycete Clades I, II and III-IV highlighted in green, blue and grey, respectively. The five AA7 isoforms from *P. infestans* and the four characterised OGOX isoforms from *Arabidopsis* are indicated using coloured circles and triangles, respectively. Bootstrap values are indicated by numbers. Tree branches highlighted in pink represent all 27 *Arabidopsis* AA7s retrieved from InterPro. Uncoloured tree branches are not part of Clades I to IV and correspond to AA7s from phytopathogenic Ascomycota (*Gibberella zeae*, *Pyricularia oryzae*) and Basidiomycota (*Puccinia graminis*, *Rhizoctonia solani*), retrieved from InterPro (see Methods for more details).
