# Supplementary material for: Oomycetes manipulate plant innate immunity through galacturonide oxidases
Source: Nat Commun. 2025 Oct 20;16:9093. doi: 10.1038/s41467-025-64189-1 (PMC12537922; doi:10.1038/s41467-025-64189-1)
Supplement: Supplementary file 2 — Description of Additional Supplementary Files [file 41467_2025_64189_MOESM2_ESM.pdf]

Description of Additional Supplementary Files:

Supplementary Data 1: RT-qPCR of *PiAA7A-E* in *P. infestans* infecting potato leaves at 24, 48 and 72 hpi.

Supplementary Data 2: Summary of kinetic parameters for *PiAA7A-D* (calculated using Prism).

Supplementary Data 3: RT-qPCR of *PiAA7A* and mScarlet tagged *PiAA7A* during infection of *Nicotiana benthamiana*.

Supplementary Data 4: RT-qPCR of *PiAA7A-E* in silenced P. infestans lines.
