# Supplementary material for: Oomycetes manipulate plant innate immunity through galacturonide oxidases
Source: Nat Commun. 2025 Oct 20;16:9093. doi: 10.1038/s41467-025-64189-1 (PMC12537922; doi:10.1038/s41467-025-64189-1)
Supplement: Supplementary file 7 — Reporting Summary [file 41467_2025_64189_MOESM7_ESM.pdf]

nature portfolio

Corresponding author(s): Federico Sabbadin

Last updated by author(s): Jul 3, 2025

Reporting Summary

Nature Portfolio wishes to improve the reproducibility of the work that we publish. This form provides structure for consistency and transparency in reporting. For further information on Nature Portfolio policies, see our Editorial Policies and the Editorial Policy Checklist.

Statistics

For all statistical analyses, confirm that the following items are present in the figure legend, table legend, main text, or Methods section.

n/a	Confirmed	
[ ]	[x]	The exact sample size (*n*) for each experimental group/condition, given as a discrete number and unit of measurement
[ ]	[x]	A statement on whether measurements were taken from distinct samples or whether the same sample was measured repeatedly
[ ]	[x]	The statistical test(s) used AND whether they are one- or two-sided<br>*Only common tests should be described solely by name; describe more complex techniques in the Methods section.*
[x]	[ ]	A description of all covariates tested
[x]	[ ]	A description of any assumptions or corrections, such as tests of normality and adjustment for multiple comparisons
[ ]	[x]	A full description of the statistical parameters including central tendency (e.g. means) or other basic estimates (e.g. regression coefficient) AND variation (e.g. standard deviation) or associated estimates of uncertainty (e.g. confidence intervals)
[ ]	[x]	For null hypothesis testing, the test statistic (e.g. *F*, *t*, *r*) with confidence intervals, effect sizes, degrees of freedom and *P* value noted<br>*Give P values as exact values whenever suitable.*
[x]	[ ]	For Bayesian analysis, information on the choice of priors and Markov chain Monte Carlo settings
[x]	[ ]	For hierarchical and complex designs, identification of the appropriate level for tests and full reporting of outcomes
[ ]	[x]	Estimates of effect sizes (e.g. Cohen's *d*, Pearson's *r*), indicating how they were calculated

Our web collection on statistics for biologists contains articles on many of the points above.

Software and code

Policy information about availability of computer code

Data collection	MxPro QPCR. Bruker Compass 3.4. StepOne 2.0. Tablet 1.19.09.03. OMERO.web 5.14.0. SPECTROstar (R) Nano software version 6.20. UNICORN start 1.3. Chromeleon 7.
Data analysis	MxPro QPCR. Bruker Compass 3.4. StepOne 2.0. StepOne Software v2.3. Tablet 1.19.09.03. OMERO.web 5.14.0. MARS program version 5.02 R3. Chromeleon 7.

For manuscripts utilizing custom algorithms or software that are central to the research but not yet described in published literature, software must be made available to editors and reviewers. We strongly encourage code deposition in a community repository (e.g. GitHub). See the Nature Portfolio guidelines for submitting code & software for further information.

Data

Policy information about availability of data

All manuscripts must include a data availability statement. This statement should provide the following information, where applicable:

- Accession codes, unique identifiers, or web links for publicly available datasets
- A description of any restrictions on data availability
- For clinical datasets or third party data, please ensure that the statement adheres to our policy

All data are available in the main text or the supplementary materials.

Research involving human participants, their data, or biological material

Policy information about studies with human participants or human data. See also policy information about sex, gender (identity/presentation), and sexual orientation and race, ethnicity and racism.

Reporting on sex and gender	N/A
Reporting on race, ethnicity, or other socially relevant groupings	N/A
Population characteristics	N/A
Recruitment	N/A
Ethics oversight	N/A

Note that full information on the approval of the study protocol must also be provided in the manuscript.

Field-specific reporting

Please select the one below that is the best fit for your research. If you are not sure, read the appropriate sections before making your selection.

☒ Life sciences ☐ Behavioural & social sciences ☐ Ecological, evolutionary & environmental sciences

For a reference copy of the document with all sections, see nature.com/documents/nr-reporting-summary-flat.pdf

Life sciences study design

All studies must disclose on these points even when the disclosure is negative.

Sample size	At least three independent replicates were collected/measured for each experiment or time point across the manuscript, with the only exception of P. infestans line IR10 where two replicates were used for lesion size and RT-qPCR. Three separate mScarlet lines (each one with one replicate) were used for the native promotor expression experiment.
Data exclusions	N/A
Replication	At least three independent replicates were collected/measured for each experiment or time point across the manuscript, with the only exception of P. infestans line IR10 where two replicates were used for lesion size and RT-qPCR. Three separate mScarlet lines (each one with one replicate) were used for the native promotor expression experiment.
Randomization	*Describe how samples/organisms/participants were allocated into experimental groups. If allocation was not random, describe how covariates were controlled OR if this is not relevant to your study, explain why.*
Blinding	*Describe whether the investigators were blinded to group allocation during data collection and/or analysis. If blinding was not possible, describe why OR explain why blinding was not relevant to your study.*

Reporting for specific materials, systems and methods

We require information from authors about some types of materials, experimental systems and methods used in many studies. Here, indicate whether each material, system or method listed is relevant to your study. If you are not sure if a list item applies to your research, read the appropriate section before selecting a response.

Materials & experimental systems

n/a	Involved in the study	
☐	☒	Antibodies
☒	☐	Eukaryotic cell lines
☒	☐	Palaeontology and archaeology
☐	☒	Animals and other organisms
☒	☐	Clinical data
☒	☐	Dual use research of concern
☐	☒	Plants

Methods

n/a	Involved in the study	
☒	☐	ChIP-seq
☒	☐	Flow cytometry
☒	☐	MRI-based neuroimaging

Antibodies

Antibodies used	Mouse α-RFP antibody (6G6 ProteinTech), goat anti-mouse antibody conjugated to IRdye 800CW (LICORbio), rabbit α-histone H3

	antibody (Abcam) and IR dye 680RD-conjugated goat anti-rabbit antibody (LICORbio)
Validation	The α-RFP (mouse monoclonal, 6G6 ProteinTech) and α-histone H3 (rabbit polyclonal, Abcam) antibodies were validated based on expected specificity and molecular weight detection. The α-RFP antibody detected the mCherry fusion protein in both mycelia and culture filtrate, consistent with secretion of the tagged protein. The α-histone H3 antibody detected a band only in mycelial extracts, not in the culture filtrate, confirming nuclear specificity and supporting the absence of cell lysis. These findings are shown for three biological replicates in the immunoblot data provided in the manuscript.

Animals and other research organisms

Policy information about studies involving animals; ARRIVE guidelines recommended for reporting animal research, and Sex and Gender in Research

Laboratory animals	*For laboratory animals, report species, strain and age OR state that the study did not involve laboratory animals.*
Wild animals	*Provide details on animals observed in or captured in the field; report species and age where possible. Describe how animals were caught and transported and what happened to captive animals after the study (if killed, explain why and describe method; if released, say where and when) OR state that the study did not involve wild animals.*
Reporting on sex	*Indicate if findings apply to only one sex; describe whether sex was considered in study design, methods used for assigning sex. Provide data disaggregated for sex where this information has been collected in the source data as appropriate; provide overall numbers in this Reporting Summary. Please state if this information has not been collected. Report sex-based analyses where performed, justify reasons for lack of sex-based analysis.*
Field-collected samples	*For laboratory work with field-collected samples, describe all relevant parameters such as housing, maintenance, temperature, photoperiod and end-of-experiment protocol OR state that the study did not involve samples collected from the field.*
Ethics oversight	*Identify the organization(s) that approved or provided guidance on the study protocol, OR state that no ethical approval or guidance was required and explain why not.*

Note that full information on the approval of the study protocol must also be provided in the manuscript.

Plants

Seed stocks	Seed stocks were sourced at the James Hutton Institute. Nicotiana benthamiana WT. Arabidopsis thaliana Col-0. Solanum lycopersicum MicroTom. Solanum tuberosum cultivar Maris Piper.
Novel plant genotypes	N/A
Authentication	N/A
